# HAUS5 defines a mitogen-activated protein kinase/extracellular signal-regulated kinase-dependent epithelial–mesenchymal plasticity program and therapeutic vulnerability to Belinostat in clear cell renal cell carcinoma

**DOI:** 10.1016/j.tranon.2026.103107

**Published:** 2026-11-06

**Authors:** Youwei Chi, Yanfei Li, Bin Yang, Guixin Ding, Chao Tang, Fengze Sun, Qian Li, Renxiang Zhang, Jian Ma, Hongchang Zhang, Jitao Wu, Yongwei Li

**Affiliations:** a Yantai Yuhuangding Hospital, Qingdao University, Yantai, Shandong, China; b Yantai Affiliated Hospital of Binzhou Medical University, Yantai, Shandong, China; c College of Life Sciences, University of Chinese Academy of Sciences, Beijing, 100049, China

**Keywords:** ccRCC, HAUS5, Epithelial–mesenchymal transition, Macrophage polarization, Belinostat

## Abstract

Clear cell renal cell carcinoma (ccRCC) is characterized by high aggressiveness and limited therapeutic options. However, the molecular determinants driving ccRCC progression and therapeutic vulnerability remain incompletely defined. Through integrated bulk and single-cell transcriptomic analyses, we identified HAUS5 as a tumor-enriched factor that was highly expressed in ccRCC tissues and tumor-derived epithelial cells. Elevated HAUS5 expression was associated with adverse clinicopathological features and poor overall survival. Functional assays showed that HAUS5 depletion suppressed ccRCC cell proliferation, migration, invasion, and xenograft tumor growth. Mechanistically, HAUS5 was associated with MAPK/ERK signaling activation and epithelial–mesenchymal transition, whereas EGF partially rescued the inhibitory effects induced by HAUS5 knockdown. In addition, drug sensitivity analysis identified Belinostat as a candidate compound associated with HAUS5 expression, and functional experiments showed that Belinostat attenuated HAUS5-associated malignant phenotypes. Collectively, these findings suggest that HAUS5 contributes to ccRCC progression through a MAPK/ERK-associated EMT program and may be linked to differential sensitivity to Belinostat.

## Highlights

• HAUS5 is upregulated in ccRCC and associated with poor clinical outcome.
• HAUS5 promotes ccRCC proliferation, migration, invasion, and tumor growth.
• HAUS5 promotes MAPK/ERK-associated EMT in ccRCC.
• HAUS5 regulates MIF-associated macrophage remodeling in ccRCC.
• Belinostat suppresses HAUS5-driven EMT and malignant phenotypes.

## Graphical abstract

**Figure:**
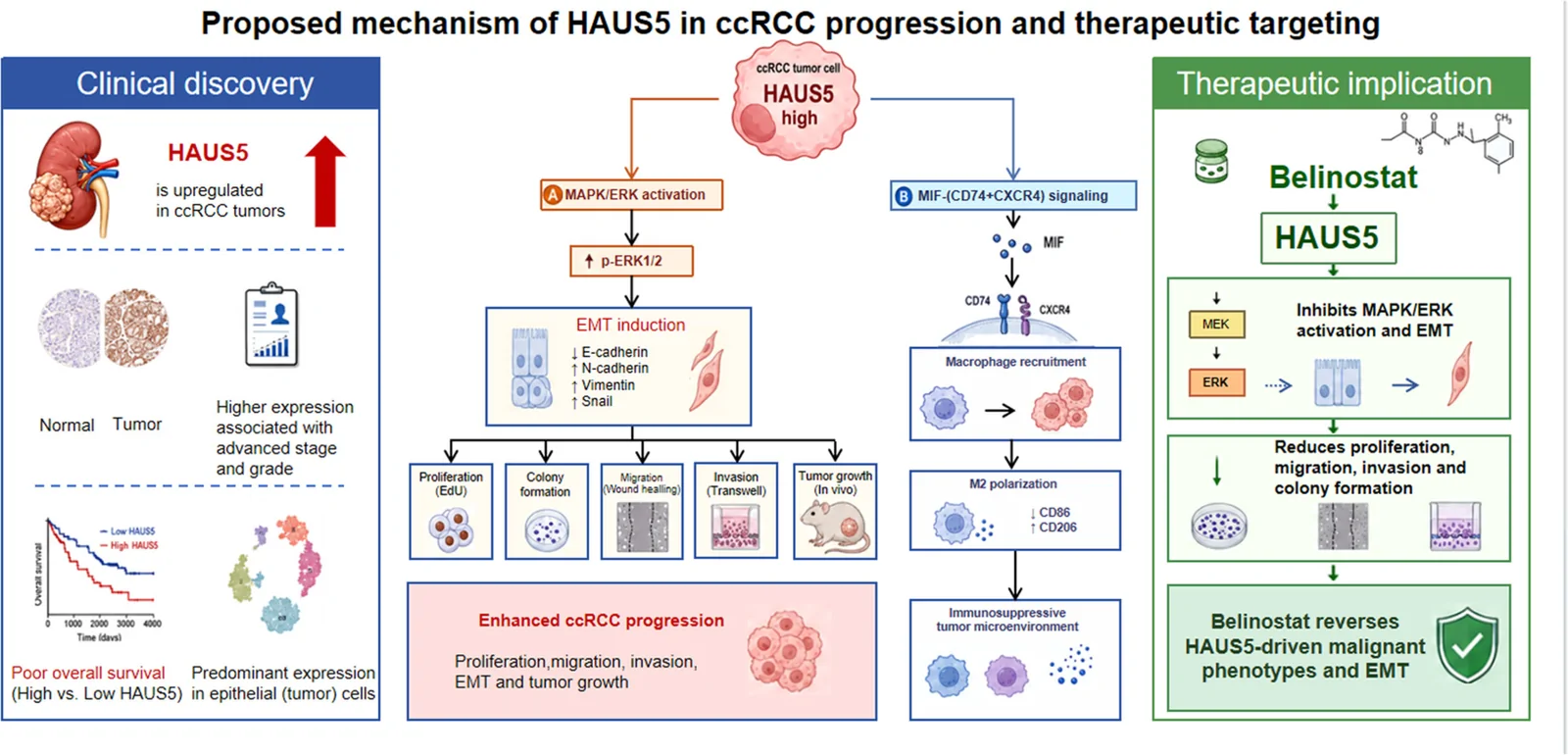

## Introduction

Renal cell carcinoma (RCC) stands as the most prevalent malignancy of the kidney and is ranked among the top ten most frequently diagnosed cancers in the United States [1]. By 2026, it is projected that there will be 80,450 new cases of RCC and 15,160 RCC-related deaths in the United States [2]. Remarkably, around one-third of RCC cases are diagnosed at advanced stages, where the therapeutic responses to the current standard of care interventions are still less than optimal [3]. ccRCC, the predominant histological subtype, makes up 75–85% of all RCC cases [[4], [5], [6]]. Currently, the field of ccRCC still lacks accurate early diagnostic markers and reliable prognostic assessment indicators [7,8].

HAUS5, also referred to as Dgt5 or KIAA0841, is a member of the HAUS family [9]. It is localized to the interphase centrosome and mitotic spindle microtubules [9]. Multiple studies have shown that the aberrant expression of HAUS5 leads to an elevated mitotic index, a notably increased proportion of abnormal spindles, and centrosome fragmentation, thus facilitating tumorigenesis [10]. Moreover, the dysregulation of HAUS5 has been associated with the development and advancement of several malignancies, such as breast cancer and hepatocellular carcinoma [11,12].

Epithelial-mesenchymal transition (EMT) is a key driver of tumor invasion and metastasis, during which epithelial cells lose polarity and adhesion, downregulate E-cadherin, upregulate vimentin and N-cadherin, and acquire invasive mesenchymal traits [13,14]. The MAPK pathway, central to intracellular signal transduction, mediates extracellular stimuli, with ERK1/2 as its core effector [15]. ERK1/2 phosphorylates transcription factors (e.g., Snail, Twist), triggering these EMT changes [[16], [17], [18]]. Consequently, ERK-driven EMT promotes metastasis and confers resistance to radiotherapy and chemotherapy. Targeting this pathway to reverse EMT is a promising strategy to improve cancer treatment. Histone deacetylases (HDACs) are important epigenetic regulators of tumor-cell proliferation, survival, and phenotypic plasticity. Previous studies in renal carcinoma have shown that HDAC inhibition can suppress tumor-cell growth and survival and modulate EMT-associated cellular plasticity [19]. In particular, HDAC1 and HDAC2 have been implicated in renal carcinoma cell growth and survival, whereas inhibition of class I HDACs has been reported to interfere with EMT-related and metastasis-associated processes [19,20]. In addition, systematic analyses of HDAC family members in clear cell renal cell carcinoma have suggested potential prognostic and biological relevance for several HDAC isoforms, including HDAC8, HDAC10, and HDAC11 [21,22]. Belinostat is a pan-HDAC inhibitor that acts on zinc-dependent HDACs and has shown antitumor activity in several malignancies. The major HDAC classes related to Belinostat and their reported relevance to renal cancer and EMT are summarized in Table 1. However, whether HAUS5 expression is associated with differential responsiveness to Belinostat in ccRCC remains unclear. In this study, we precisely evaluated the expression level of HAUS5 in ccRCC and its prognostic significance. Subsequently, both in vitro and in vivo experiments were carried out to validate the role of HAUS5 in ccRCC. Moreover, the potential role of HAUS5 in ccRCC progression and its underlying mechanism were further investigated.

**Table 1:** Histone deacetylase classes targeted by Belinostat and their potential relevance to renal cancer.

HDAC class	HDAC members	Relationship to Belinostat	Potential relevance to RCC/EMT
Class I	HDAC1, HDAC2, HDAC3, HDAC8	Zinc-dependent HDACs inhibited by Belinostat	HDAC1/2 contribute to renal carcinoma cell growth and survival, class I HDAC inhibition has been reported to modulate EMT-associated cellular plasticity
Class IIa	HDAC4, HDAC5, HDAC7, HDAC9	Zinc-dependent HDACs within the broader inhibitory spectrum of pan-HDAC inhibition; isoform potency varies	Direct RCC-specific mechanistic evidence remains comparatively limited
Class IIb	HDAC6, HDAC10	Zinc-dependent HDACs within the inhibitory spectrum of Belinostat	HDAC10 has been reported to have potential prognostic and biological relevance in ccRCC
Class III	SIRT1–SIRT7	Not classical targets of Belinostat	NAD⁺-dependent deacetylases with a distinct catalytic mechanism
Class IV	HDAC11	Zinc-dependent HDAC; reported sensitivity to pan-HDAC inhibition varies among assays	HDAC11 has been reported to have potential prognostic and tumor-microenvironment relevance in ccRCC

**Note:** Belinostat is a hydroxamate-based pan-HDAC inhibitor targeting zinc-dependent HDACs in classes I, II, and IV, whereas class III sirtuins are NAD⁺-dependent and are not considered classical targets of Belinostat. Reported inhibitory potency varies among individual HDAC isoforms and experimental assay systems.

## Results

### HAUS5 is tumor-specifically upregulated and clinically relevant in ccRCC

To determine the clinical significance of HAUS5 in clear cell renal cell carcinoma (ccRCC), we first evaluated its expression pattern across multiple cancer types. TCGA pan-cancer analysis showed that HAUS5 was upregulated in several malignancies compared with corresponding normal tissues, with a marked increase observed in ccRCC (Fig. 1A). Further analysis of the ccRCC cohort, using both unpaired and paired samples, confirmed that HAUS5 expression was significantly higher in tumor tissues than in normal kidney tissues (Fig. 1B, C).

**Fig. 1:**
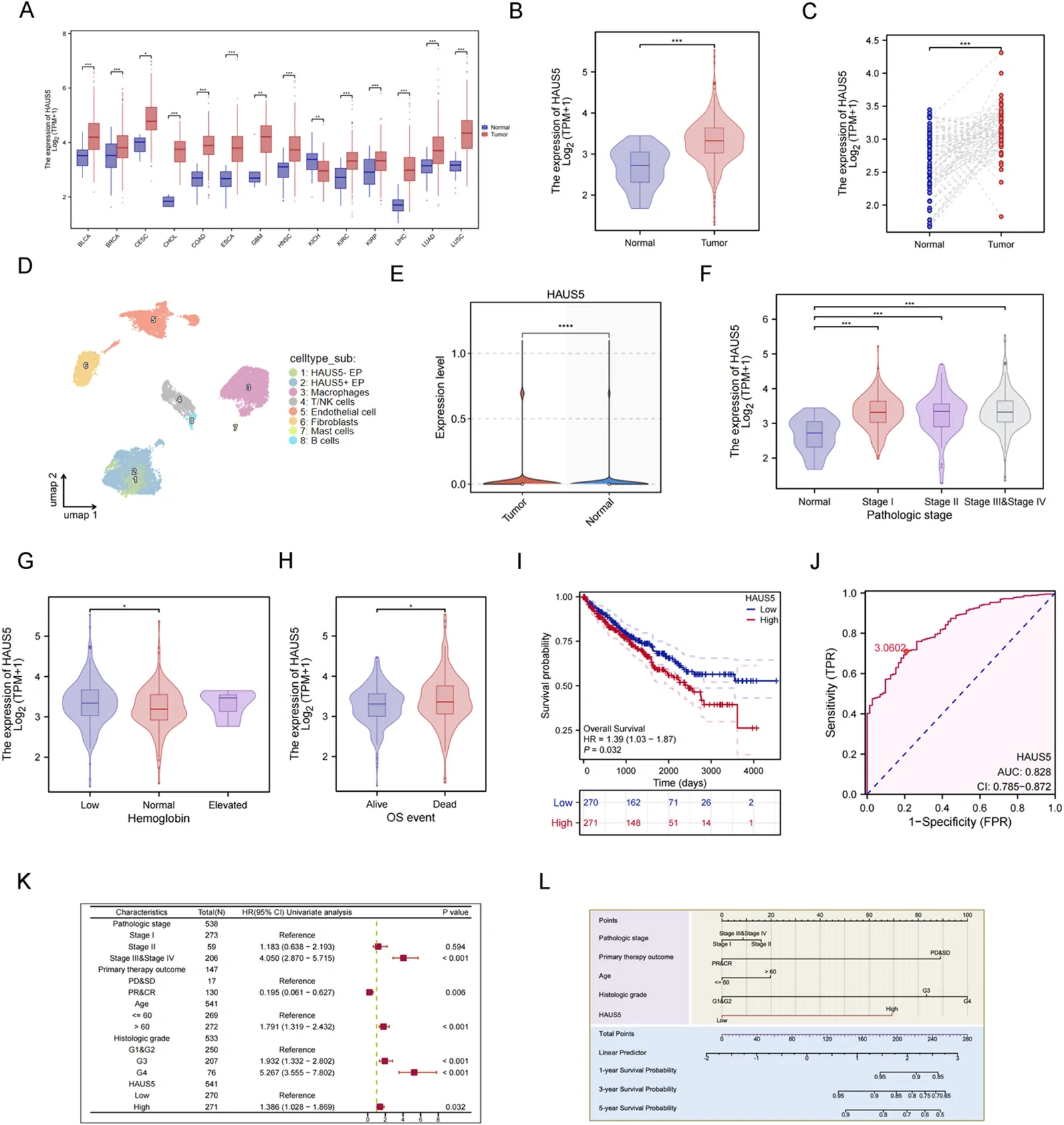
High HAUS5 expression correlated with disease progression and reduced survival in ccRCC. (A) Expression differences of HAUS5 in various cancers in TCGA. (B) Transcriptional level differences of HAUS5 between ccRCC samples and non-tumor renal tissues. (C) mRNA expression of HAUS5 in paired samples of ccRCC and adjacent normal renal tissues. (D) UMAP plot showing the major cell populations identified from the GSE159115 single-cell RNA-sequencing dataset. (E) Single-cell analysis displaying HAUS5 expression patterns in malignant epithelial cells and adjacent normal epithelial cells. (F–H) Correlations of HAUS5 expression with pathological stage, hemoglobin level, and survival outcome in patients with ccRCC. (I) Kaplan–Meier plot showing overall survival differences in ccRCC patients stratified by HAUS5 expression. (J) Receiver operating characteristic curve verified HAUS5 diagnostic efficacy in ccRCC. (K) Forest plot based on univariate Cox regression analysis showing the prognostic significance of HAUS5 and relevant clinical variables. (L) Nomogram model was established to calculate survival probability of affected patients. Data are presented as mean ± SD. *P< 0.05, **P < 0.01, ***P < 0.001, ****P < 0.0001.

To further define the cellular distribution of HAUS5 in ccRCC, we analyzed the GSE159115 single-cell RNA sequencing dataset, which included 19,006 cells. Unsupervised clustering identified eight major cell populations (Fig. 1D). HAUS5 expression was mainly detected in epithelial cells, and tumor-derived epithelial cells exhibited stronger HAUS5 signals than normal epithelial cells (Fig. 1E). These findings suggested that HAUS5 upregulation in ccRCC was primarily associated with malignant epithelial compartments.

We next examined the relationship between HAUS5 expression and clinicopathological characteristics. Higher HAUS5 expression was associated with advanced pathological stage, abnormal hemoglobin status, and death events (Fig. 1F-H). Kaplan-Meier survival analysis showed that patients with high HAUS5 expression had significantly worse overall survival than those with low HAUS5 expression (Fig. 1I). Receiver operating characteristic curve analysis further indicated that HAUS5 had diagnostic potential for distinguishing ccRCC tissues from normal kidney tissues (Fig. 1J). In addition, univariate Cox regression analysis supported the prognostic relevance of HAUS5, and a nomogram integrating HAUS5 expression with clinicopathological variables was constructed to estimate 1-, 3-, and 5-year survival probabilities (Fig. 1K, L). Together, these results indicate that HAUS5 is upregulated in ccRCC and is closely associated with disease progression and poor clinical outcome.

### HAUS5 promotes malignant phenotypes of ccRCC cells in vitro

To validate HAUS5 dysregulation in ccRCC and investigate its biological function, we examined HAUS5 expression in clinical specimens and cell models. Immunohistochemical staining showed stronger HAUS5 positivity in ccRCC tumor tissues than in adjacent normal kidney tissues, with three representative paired cases shown in Fig. 2A. Consistently, qRT-PCR analysis of 12 paired clinical samples demonstrated that HAUS5 mRNA expression was significantly elevated in ccRCC tissues compared with matched adjacent non-tumor tissues (Fig. 2B). Western blot analysis of 5 paired samples further confirmed increased HAUS5 protein abundance in tumor tissues (Fig. 2C, E). In line with these findings, multiple ccRCC cell lines displayed higher HAUS5 expression than the normal renal tubular epithelial cell line HK-2 (Fig. 2D, F).

**Fig. 2:**
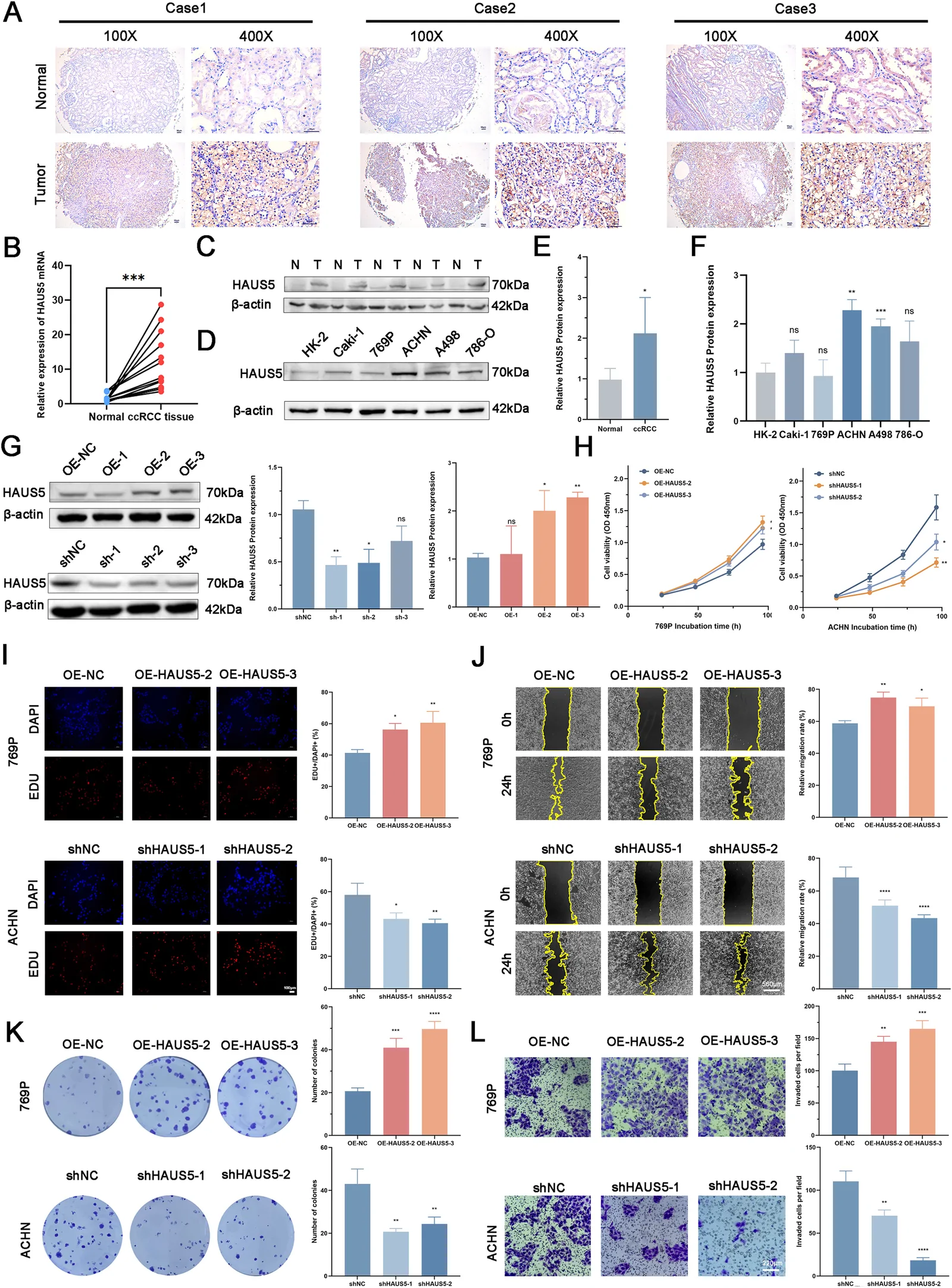
Aberrant HAUS5 expression facilitates the malignant biological behaviors of ccRCC cells in vitro. (A) Representative IHC staining of HAUS5 in ccRCC and adjacent non-tumor renal tissues; three paired cases are shown. (B) qRT-PCR analysis of HAUS5 mRNA expression in 12 paired ccRCC and adjacent non-tumor renal tissue samples (n = 12 pairs). (C) Western blot analysis of HAUS5 protein expression in 5 paired ccRCC and adjacent non-tumor renal tissue samples (n = 5 pairs). (D) Expression profiling of HAUS5 protein in renal carcinoma cell lines versus normal HK-2 epithelial cells. (E, F) Quantitative analysis of HAUS5 expression in renal tissues and renal cell lines, respectively. (G) Western blot validation of independent HAUS5 overexpression constructs in 769P cells and knockdown constructs in ACHN cells. (H) CCK-8 assay evaluating cell proliferation in 769P cells expressing OE-NC, OE-HAUS5–2, or OE-HAUS5–3 and in ACHN cells expressing shNC, shHAUS5–1, or shHAUS5–2. (I) EdU assay assessing proliferation in the indicated independent overexpression and knockdown groups. (J) Wound-healing assay evaluating cell migration in the indicated groups. (K) Colony formation assay assessing clonogenic growth in the indicated groups. (L) Transwell invasion assay evaluating invasive capacity in the indicated groups. Data are presented as mean ± SD. *P < 0.05, **P < 0.01, ***P < 0.001, ****P < 0.0001.

Based on endogenous HAUS5 expression levels, three independent HAUS5 knockdown constructs were evaluated in ACHN cells and three HAUS5 overexpression constructs were evaluated in 769P cells. Western blotting confirmed efficient HAUS5 modulation by shHAUS5–1 and shHAUS5–2 in ACHN cells and by OE-HAUS5–2 and OE-HAUS5–3 in 769P cells (Fig. 2G). Key functional assays performed with these independent constructs showed consistent phenotypic changes, with HAUS5 overexpression enhancing and HAUS5 knockdown suppressing ccRCC cell proliferation, migration, and invasion (Fig. 2H-L). These results demonstrate that HAUS5 promotes the aggressive behavior of ccRCC cells in vitro.

### HAUS5 silencing suppresses xenograft growth and alters EMT-related marker expression in vivo

To determine whether HAUS5 contributes to ccRCC tumor growth in vivo, we established a nude mouse subcutaneous xenograft model. Compared with the shNC group, HAUS5 depletion markedly inhibited xenograft growth, as shown by smaller excised tumors and reduced tumor weight in the shHAUS5 group (Fig. 3A-C). Tumor growth curves further showed that HAUS5 knockdown slowed tumor volume expansion, whereas mouse body weight remained comparable between the two groups (Fig. 3D, E).

**Fig. 3:**
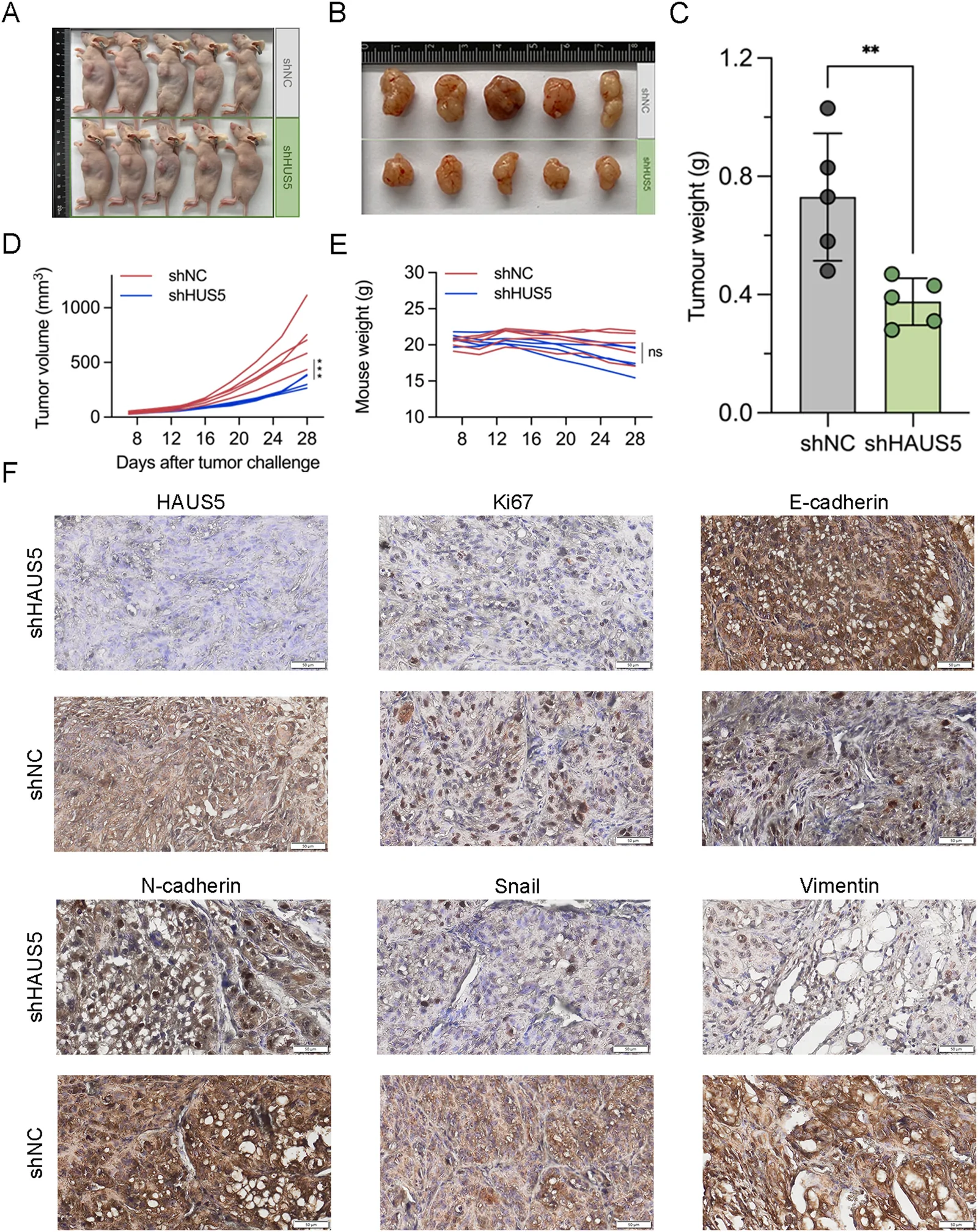
Silencing HAUS5 restrains in vivo tumor growth and remodels the expression of EMT-related proteins. (A) Morphological presentation of subcutaneous xenograft tumors derived from shNC and shHAUS5 stably transfected cells in nude mice. (B) Gross morphology of excised xenograft tumors collected from the indicated experimental groups. (C) Quantitative comparison of tumor weight between the control and HAUS5 silencing groups. (D) Tumor volume curves recorded during the xenograft experiment. (E) Dynamic changes in body weight of experimental mice were recorded during animal observation. (F) Typical IHC staining results of HAUS5, Ki67, E-cadherin, N-cadherin, Snail and Vimentin in subcutaneous tumor tissues. Data are presented as mean ± SD. ns, not significant; **P < 0.01, ***P < 0.001.

Immunohistochemical analysis of xenograft sections showed that HAUS5 and Ki67 expression levels were reduced in tumors derived from HAUS5-silenced cells. Moreover, HAUS5 depletion increased E-cadherin expression and decreased the expression of N-cadherin, Snail, and Vimentin (Fig. 3F). These findings indicate that HAUS5 knockdown suppresses ccRCC tumor growth in vivo and is accompanied by reduced proliferative activity and reversal of EMT-related marker expression.

### HAUS5 is associated with MAPK/ERK signaling activation and EMT regulation in ccRCC

To explore the potential mechanism underlying HAUS5-mediated malignant progression in ccRCC, we performed enrichment and correlation analyses of HAUS5-associated genes. Gene Ontology enrichment analysis revealed that these genes were mainly enriched in biological processes related to cell adhesion, regulation of apoptotic signaling, leukocyte proliferation, and regulation of the MAPK cascade (Fig. 4A). Pan-cancer pathway correlation analysis showed that HAUS5 expression was significantly associated with the MAPK signaling pathway in ccRCC (Fig. 4B, C). Moreover, HAUS5 expression correlated with key MAPK/ERK pathway components and EMT-related markers, suggesting a potential link between HAUS5, MAPK/ERK activation, and EMT phenotypes in ccRCC (Fig. 4D-F).

**Fig. 4:**
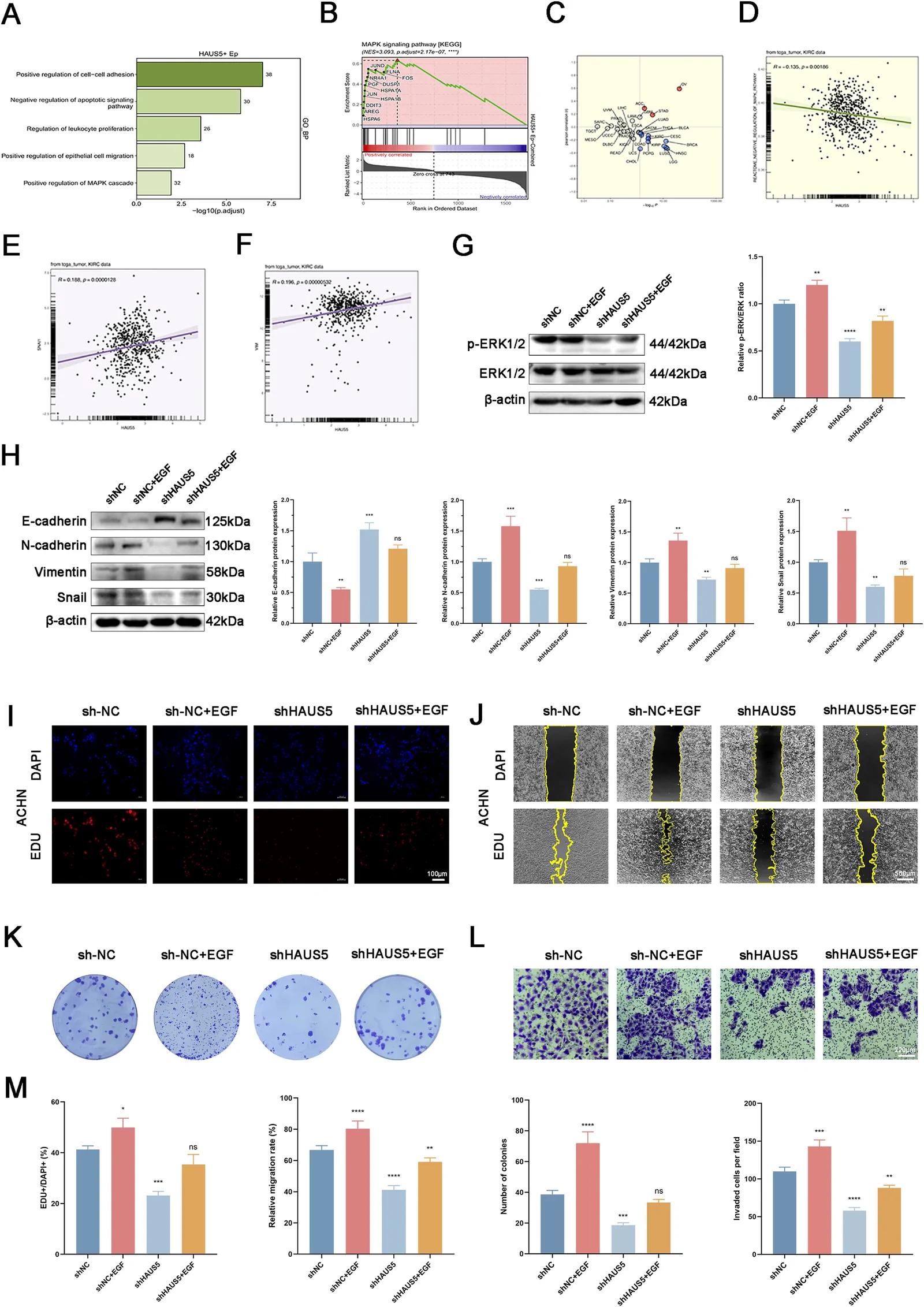
HAUS5-associated malignant phenotypes involved MAPK/ERK signaling and EMT-related changes. (A) GO-BP enrichment analysis of HAUS5-associated genes in HAUS5-positive epithelial cells. (B) GSEA showing enrichment of the MAPK signaling pathway. (C) Pan-cancer correlation analysis revealing the association between HAUS5 expression and MAPK signaling activity. (D–F) Correlation analyses between HAUS5 expression and MAPK/ERK- or EMT-related markers in ccRCC. (G) Western blot analysis of ERK1/2 phosphorylation in ACHN cells under four conditions: shNC, shNC + EGF, shHAUS5, and shHAUS5 + EGF. (H) Western blot analysis of EMT-related protein expression in the same four groups. (I) EdU assay evaluating cell proliferation. (J) Wound-healing assay assessing cell migration. (K) Colony formation assay evaluating clonogenic growth. (L) Transwell invasion assay assessing invasive capacity. (M) Quantitative analysis of the corresponding functional assays. Data are presented as mean ± SD. *P < 0.05, **P < 0.01, ***P < 0.001, ****P < 0.0001.

To experimentally assess the involvement of MAPK/ERK signaling, rescue experiments were performed in ACHN cells using four parallel conditions: shNC, shNC + EGF, shHAUS5, and shHAUS5 + EGF. Western blotting showed that HAUS5 knockdown reduced ERK1/2 phosphorylation and induced corresponding changes in EMT-related proteins, whereas EGF treatment partially restored ERK activation and EMT-associated protein expression in HAUS5-depleted cells (Fig. 4G–H). Consistently, functional assays demonstrated that HAUS5 knockdown suppressed cell proliferation, colony formation, migration, and invasion, while EGF treatment partially rescued these inhibitory effects (Fig. 4I–M, Supplementary Fig. 1A). These findings support the involvement of MAPK/ERK signaling in HAUS5-mediated EMT and malignant phenotypes in ccRCC cells.

### HAUS5 regulates macrophage recruitment, phagocytosis, and M2-like polarization in the ccRCC microenvironment

GSEA showed that high HAUS5 expression was associated with enrichment of the negative regulation of leukocyte activation pathway, suggesting that HAUS5 may be involved in immune microenvironment remodeling in ccRCC (Fig. 5A). Cell-cell communication analysis further indicated potential interactions between HAUS5-positive epithelial cells and macrophages. In particular, HAUS5-positive tumor epithelial cells appeared to communicate with macrophages through the MIF-CD74/CXCR4 axis (Fig. 5B-F). To validate MIF as a potential soluble mediator, its secretion in the conditioned medium was assessed by ELISA. HAUS5 overexpression significantly increased MIF secretion in 769P cells, whereas HAUS5 knockdown reduced MIF secretion in ACHN cells (Supplementary Fig. 1B, C). These findings were consistent with the predicted involvement of the MIF–CD74/CXCR4 signaling axis and suggested that HAUS5 expression positively regulates tumor cell-derived MIF secretion.

**Fig. 5:**
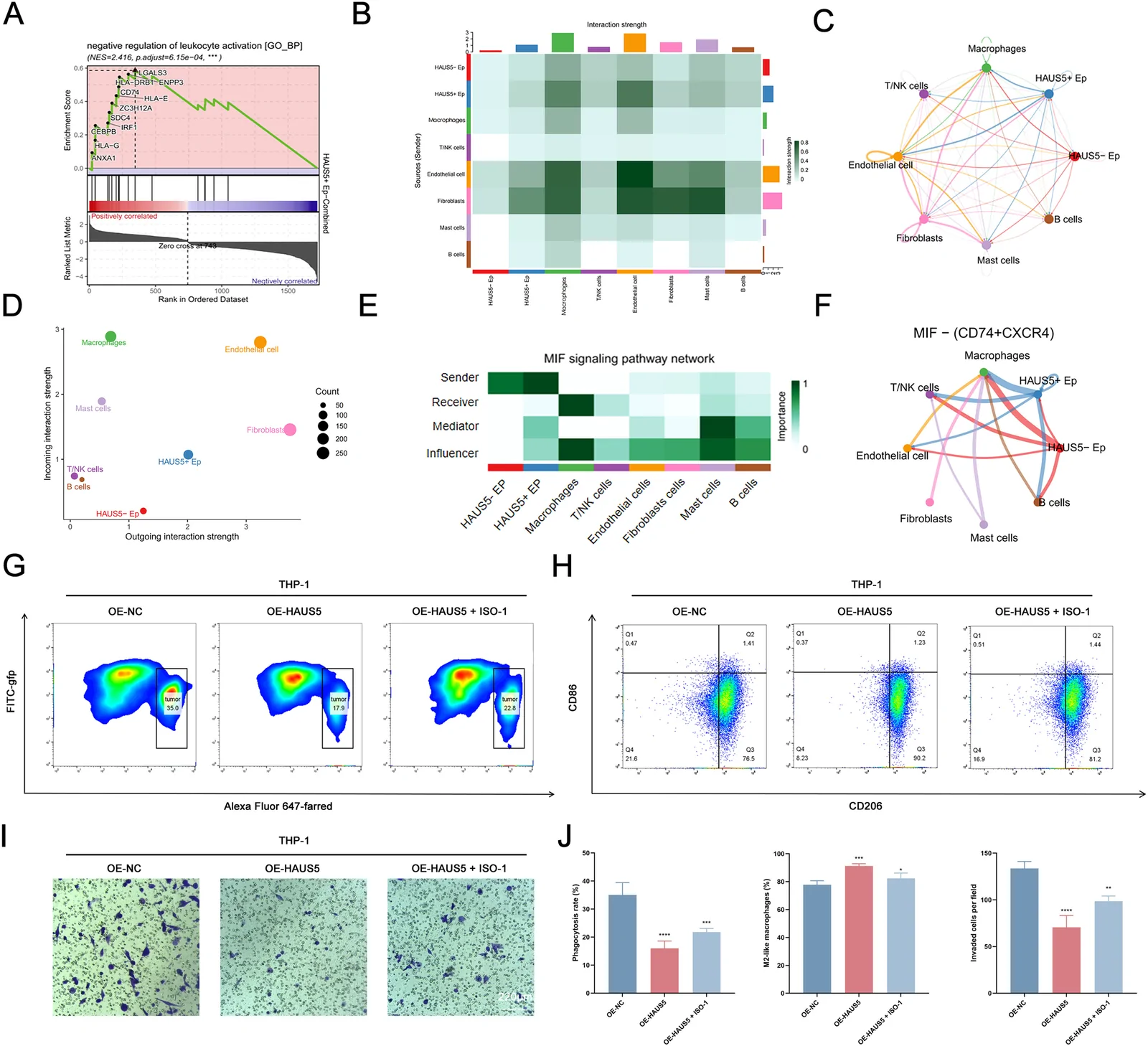
HAUS5 was involved in macrophage-mediated immune microenvironment remodeling in ccRCC. (A) Gene set enrichment analysis uncovered the enrichment pathway responsible for negative leukocyte activation in the HAUS5 high-expression subgroup. (B) Heatmap showing the overall interaction strength among major cell populations based on single-cell RNA-seq cell–cell communication analysis. (C) Circle plot showing the inferred intercellular communication network among HAUS5-positive epithelial cells, HAUS5-negative epithelial cells, macrophages, T/NK cells, endothelial cells, fibroblasts, mast cells, and B cells. (D) Scatter graph depicts the intercellular signal input and output patterns among main cellular subsets. (E) Heatmap displays the differential contribution of various cell populations to the overall MIF signaling network. (F) Circle plot showing the communication strength among major cell populations mediated by the MIF–CD74/CXCR4 ligand–receptor axis. (G) Flow cytometry analysis of macrophage phagocytosis under OE-NC, OE-HAUS5, and OE-HAUS5 + ISO-1 conditions. (H) Flow cytometry analysis of macrophage polarization markers CD86 and CD206 under the indicated conditions. (I) Transwell assay assessing macrophage recruitment under the indicated conditions.(J) Quantitative analysis of macrophage phagocytosis, M2-like macrophage proportion, and recruited macrophages. Data are presented as mean ± SD. *P < 0.05, **P < 0.01, ***P < 0.001, ****P < 0.0001.

To further validate the functional involvement of MIF signaling, conditioned media from OE-NC cells, OE-HAUS5 cells, and OE-HAUS5 cells treated with the MIF inhibitor ISO-1 were collected and applied to THP-1-derived macrophages. HAUS5 overexpression reduced macrophage phagocytic activity and recruitment while promoting M2-like polarization. Notably, ISO-1 treatment partially reversed these macrophage phenotypes, as evidenced by increased phagocytosis and recruitment and reduced M2-like polarization (Fig. 5G–J). These findings provide functional evidence that MIF signaling contributes to HAUS5-mediated regulation of macrophage behavior in ccRCC.

### Belinostat antagonizes HAUS5-driven malignant phenotypes in ccRCC cells

To identify potential therapeutic agents for HAUS5-high ccRCC, we performed drug sensitivity correlation analysis. Among the predicted compounds, Belinostat showed a strong association with HAUS5 expression and favorable predicted sensitivity (Fig. 6A). Molecular docking analysis was performed to characterize the predicted docking pattern between Belinostat and the HAUS5 protein model. A favorable docking pose was obtained for Belinostat in the HAUS5 protein model (Fig. 6B).

**Fig. 6:**
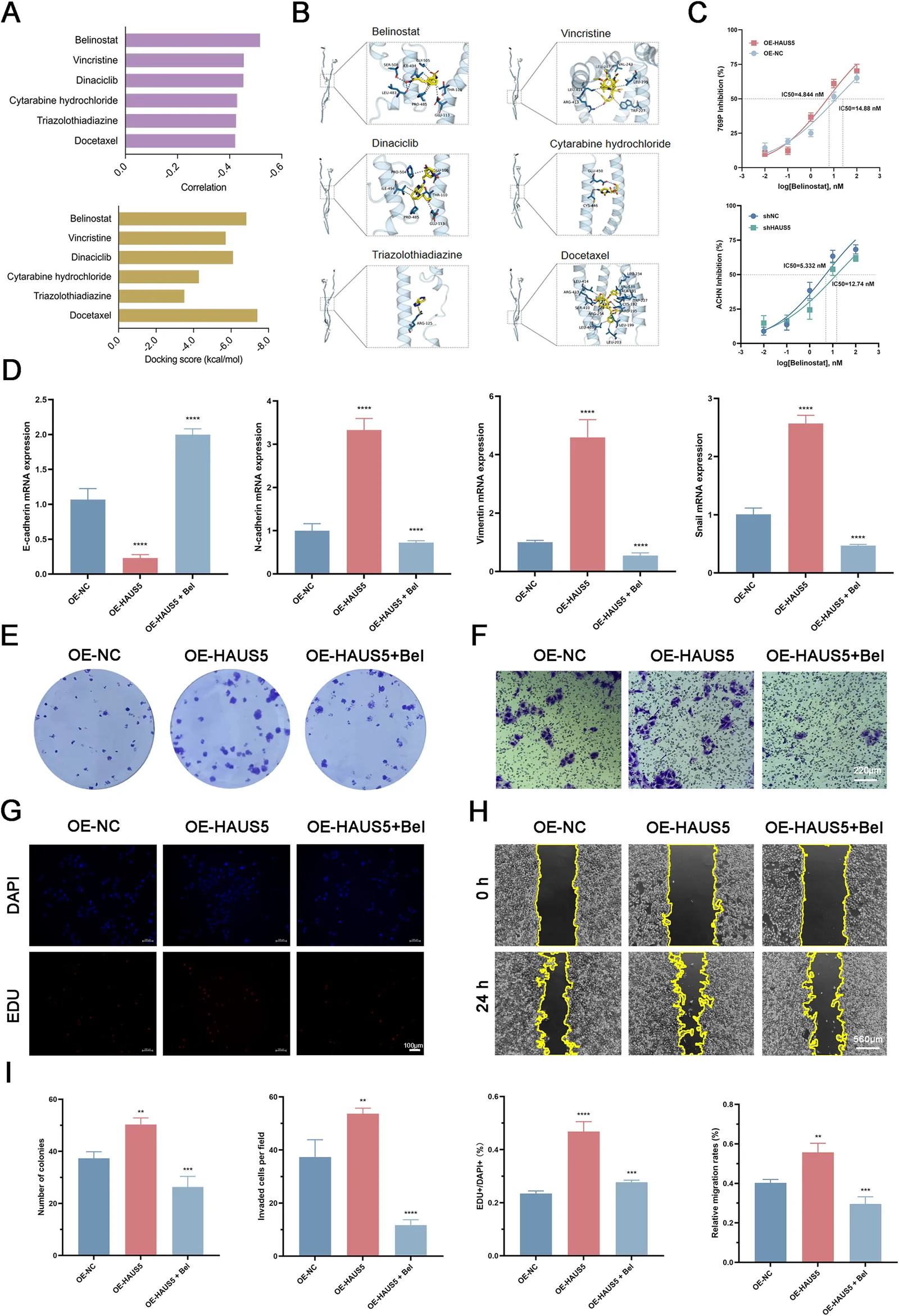
Belinostat inhibits HAUS5-mediated EMT process and malignant biological phenotypes of ccRCC cells. (A) Drug sensitivity correlation analysis identifying candidate compounds associated with HAUS5 expression. (B) Molecular docking analysis showing the predicted docking poses of candidate compounds, including Belinostat, with the HAUS5 protein model. (C) IC50 curves of Belinostat in 769P and ACHN cells with different HAUS5 expression levels. (D) qRT-PCR was performed to detect the transcription levels of EMT signature genes (E-cadherin, N-cadherin, Vimentin, Snail) in HAUS5-overexpressing ccRCC cells with or without Belinostat treatment. (E) Colony formation assay showing the long-term proliferative capacity of HAUS5-overexpressing ccRCC cells after Belinostat treatment. (F) Transwell invasion experiment evaluated the invasive behavior of HAUS5-upregulated ccRCC cells after Belinostat intervention. (G) EdU staining assay was adopted to assess the proliferative potential of HAUS5-overexpressing ccRCC cells upon Belinostat administration. (H) Wound scratch assay was conducted to measure the migration capability of HAUS5-overexpressing ccRCC cells following Belinostat treatment. (I) Quantitative analysis of colony formation, Transwell invasion, EdU-positive cells, and wound-healing assays in the indicated groups. Data are presented as mean ± SD. **P < 0.01, ***P < 0.001, ****P < 0.0001.

Drug sensitivity assays showed that HAUS5 overexpression increased Belinostat sensitivity in 769P cells, whereas HAUS5 knockdown reduced Belinostat sensitivity in ACHN cells (Fig. 6C). qRT-PCR analysis further demonstrated that Belinostat counteracted HAUS5 overexpression-induced changes in EMT-related markers, as reflected by increased E-cadherin expression and decreased N-cadherin, Vimentin, and Snail expression (Fig. 6D). Consistent with these molecular changes, Belinostat treatment attenuated HAUS5 overexpression-driven increases in cell proliferation, migration, and invasion (Fig. 6E-I). These results suggest that Belinostat suppresses malignant phenotypes in HAUS5-high ccRCC cells and may partially reverse HAUS5-induced EMT.

## Discussion

This study demonstrated that HAUS5 is significantly upregulated in ccRCC tissues and is closely associated with adverse clinicopathological features and poor prognosis. Functional experiments further showed that HAUS5 promotes the malignant phenotypes of ccRCC cells, including proliferation, migration, invasion, and xenograft tumor growth. Mechanistically, HAUS5 appears to facilitate ccRCC progression, at least in part, through activation of the MAPK/ERK signaling pathway and induction of epithelial–mesenchymal transition (EMT). In addition, our findings suggest a potential relationship between HAUS5 expression and the immune microenvironment, especially macrophage-related remodeling. Drug sensitivity prediction and experimental validation further indicated that HAUS5-high ccRCC cells may exhibit therapeutic vulnerability to Belinostat. Together, these results identify HAUS5 as a potential oncogenic regulator and therapeutic target in ccRCC.

The HAUS family consists of evolutionarily conserved protein complexes that participate in the regulation of microtubule nucleation, spindle assembly, and mitotic progression. Microtubule dynamics are essential for accurate chromosome segregation and normal cell division. Disruption of microtubule organization can induce chromosomal instability, abnormal mitosis, and malignant transformation, which are common features of tumor progression [9,[23], [24], [25]]. Previous studies have mainly focused on the role of HAUS family members in centrosome function and microtubule organization. However, the clinical significance and biological function of HAUS5 in ccRCC have not been fully defined. In the present study, we found that HAUS5 was highly expressed in ccRCC tissues and that elevated HAUS5 expression predicted unfavorable survival outcomes. These findings suggest that HAUS5 may not only reflect abnormal proliferative activity but may also actively participate in ccRCC progression.

Single-cell RNA sequencing analysis further refined the cellular localization of HAUS5 in the ccRCC microenvironment. HAUS5 expression was mainly enriched in tumor-derived epithelial cells, and its expression level was higher in malignant epithelial cells than in normal renal epithelial cells. This observation supports the idea that HAUS5 upregulation is primarily associated with the tumor cell compartment rather than being a nonspecific change across all cell types. Notably, cell–cell communication analysis identified MIF–CD74/CXCR4 as a prominent signaling pathway between HAUS5-high tumor epithelial cells and macrophages. MIF is a multifunctional cytokine that has been reported to promote tumor immune escape, immune tolerance, macrophage recruitment, and inflammatory remodeling in multiple malignancies [26,27]. Consistent with this prediction, pharmacological inhibition of MIF with ISO-1 partially reversed the effects of HAUS5 overexpression on macrophage phagocytosis, M2-like polarization, and recruitment. These findings provide functional support for the involvement of MIF signaling in HAUS5-mediated macrophage remodeling in ccRCC.

To explore the molecular basis by which HAUS5 promotes ccRCC progression, we performed enrichment analysis of HAUS5-related genes. The results showed that HAUS5-associated genes were enriched in biological processes related to MAPK signaling and EMT regulation. The MAPK/ERK pathway is one of the key signaling cascades involved in tumor cell proliferation, survival, migration, and invasion. Persistent activation of ERK1/2 can induce transcriptional programs that support tumor progression and metastatic potential. EMT is another important biological process in which epithelial tumor cells lose epithelial characteristics and acquire mesenchymal features, thereby gaining stronger migratory and invasive abilities. In ccRCC, EMT is closely related to tumor aggressiveness, metastasis, drug resistance, and poor clinical outcome. Therefore, the enrichment of HAUS5-related genes in the MAPK/ERK and EMT pathways provides a plausible mechanistic explanation for the oncogenic effects of HAUS5.

Previous studies have shown that dysregulated microtubule dynamics can influence EMT by affecting intracellular signaling transduction, cytoskeletal remodeling, and ERK1/2 nuclear translocation [[28], [29], [30], [31]]. These findings are consistent with our results and suggest that HAUS5 may connect microtubule-associated regulation with MAPK/ERK-dependent EMT activation in ccRCC. In our experimental models, HAUS5 knockdown markedly reduced ERK1/2 phosphorylation and reversed EMT-related marker expression, as shown by increased E-cadherin and decreased N-cadherin, Vimentin, and Snail. These molecular changes were accompanied by reduced proliferation, migration, and invasion of ccRCC cells. Moreover, EGF-mediated activation of MAPK/ERK signaling partially rescued the inhibitory effects caused by HAUS5 silencing. These rescue experiments further support the view that MAPK/ERK signaling participates in HAUS5-mediated EMT and malignant progression. However, because EGF only partially reversed the effects of HAUS5 knockdown, HAUS5 may also regulate ccRCC progression through additional signaling pathways that require further investigation.

In addition to its direct role in tumor cells, HAUS5 may also influence the ccRCC immune microenvironment. Our results showed that HAUS5 expression was associated with immune-related pathways, including negative regulation of leukocyte activation. Functional experiments using conditioned medium further suggested that HAUS5 altered macrophage behavior. HAUS5 overexpression reduced macrophage recruitment and phagocytic activity, while increasing the proportion of CD206-positive M2-like macrophages. These findings suggest that HAUS5 may help establish an immunosuppressive microenvironment by weakening macrophage phagocytic function and promoting M2-like polarization. Since M2-like tumor-associated macrophages are generally linked to immune suppression, angiogenesis, matrix remodeling, and tumor progression, the HAUS5-associated macrophage phenotype may contribute to ccRCC aggressiveness. However, the specific soluble mediators connecting HAUS5-high tumor cells and macrophage functional changes remain to be identified.

Belinostat is a histone deacetylase inhibitor approved by the US Food and Drug Administration for the treatment of relapsed or refractory peripheral T-cell lymphoma. In recent years, Belinostat has attracted increasing attention for its potential therapeutic effects in solid tumors [32,33]. In this study, drug sensitivity analysis identified Belinostat as a candidate compound associated with HAUS5 expression. Molecular docking further suggested a potential interaction between Belinostat and HAUS5. Functionally, HAUS5 overexpression increased Belinostat sensitivity, whereas HAUS5 knockdown reduced Belinostat sensitivity. In addition, Belinostat attenuated HAUS5-induced EMT-related marker changes and suppressed HAUS5-driven proliferation, migration, and invasion. These results suggest that Belinostat may have therapeutic relevance in HAUS5-high ccRCC. However, the precise relationship between HAUS5 and Belinostat response remains to be clarified. Belinostat may attenuate HAUS5-associated malignant phenotypes in ccRCC cells.

Although our study provides bioinformatic and experimental evidence that HAUS5 promotes ccRCC progression through the MAPK/ERK-EMT axis and may be associated with Belinostat sensitivity, several limitations should be acknowledged. A major limitation of the present study is that the in vivo validation was restricted to HAUS5 knockdown xenograft models. Although both knockdown and overexpression approaches were used in vitro, an HAUS5 overexpression xenograft model was not included. Therefore, the current in vivo findings primarily support the tumor-suppressive consequences of HAUS5 depletion rather than providing complete bidirectional causal evidence for the tumor-promoting role of HAUS5. Future studies incorporating HAUS5 overexpression xenograft models will be necessary to further establish this causal relationship. Second, the nude mouse xenograft model cannot fully reproduce the complex immune microenvironment of human ccRCC, especially because immunodeficient mice are limited for evaluating tumor–immune interactions. Future studies using immunocompetent or humanized mouse models may better clarify the role of HAUS5 in immune microenvironment remodeling. Third, although our data suggest that HAUS5 is associated with macrophage recruitment, phagocytosis, and M2-like polarization, the downstream mediators responsible for these effects remain unclear. Although our findings support the involvement of MIF signaling in HAUS5-associated macrophage remodeling, the partial reversal after MIF inhibition suggests that additional soluble mediators may also contribute to this process. Finally, although HAUS5 expression was associated with differential sensitivity to Belinostat, the molecular basis of this relationship remains unclear. The molecular docking analysis was exploratory and provides only computational support for a possible structural relationship. Further biochemical and in vivo studies are needed to clarify the mechanisms underlying Belinostat responsiveness in HAUS5-high ccRCC.

In conclusion, this study identifies HAUS5 as a tumor-promoting factor in ccRCC. HAUS5 is highly expressed in ccRCC tissues and tumor epithelial cells, predicts poor clinical outcome, and promotes malignant phenotypes through MAPK/ERK-associated EMT activation. HAUS5 may also contribute to immune microenvironment remodeling by regulating macrophage behavior. Furthermore, HAUS5-high ccRCC cells may show potential vulnerability to Belinostat treatment. These findings provide new insight into the biological function of HAUS5 in ccRCC and suggest that HAUS5 may serve as a promising prognostic biomarker and therapeutic target.

## Materials and methods

### Clinical specimens and ethics

The institutional ethics board of Yantai Yuhuangding Hospital authorized the present study, which was performed in strict adherence to the Helsinki Declaration principles. Every patient involved in this research completed written informed consent forms. Tumor tissues and paired non-cancerous kidney tissues were surgically collected from patients with ccRCC who underwent radical nephrectomy and had not received preoperative chemotherapy or targeted therapy. All diagnoses were confirmed by histopathological examination. The in-house cohort comprised 12 patients with ccRCC. Paired tumor and adjacent non-tumor renal tissues from all 12 patients were used for qRT-PCR analysis, whereas 5 paired samples with sufficient protein material were used for Western blot analysis. Representative immunohistochemical images from three paired cases are shown in Fig. 2A. The basic clinicopathological characteristics of the 12 patients are summarized in Supplementary Table 1.

### Bioinformatics analysis

Gene expression profiles and clinical metadata of ccRCC were retrieved from the TCGA database. The expression landscape of HAUS5 was profiled across pan-cancer and ccRCC cohorts via paired and unpaired tumor-normal comparative analysis. Kaplan–Meier survival analysis, ROC curve testing, Cox regression models and nomogram plotting were applied to clarify the correlations of HAUS5 levels with clinicopathological characteristics, patient survival, diagnostic value and prognostic risk.

### Single-cell and cell-cell communication analysis

The GSE159115 dataset was processed in R using Seurat for quality control, normalization, clustering, and UMAP visualization. After low-quality cells were removed, the expression matrix was normalized and subjected to dimensionality reduction, clustering, and UMAP projection. Cell identities were annotated according to established lineage markers, and HAUS5 expression was compared among the annotated cell populations. Ligand-receptor analysis was performed to infer intercellular signaling, with particular focus on the MIF-CD74/CXCR4 communication axis involving HAUS5-positive epithelial cells and macrophage populations.

### Enrichment and pathway correlation analysis

We screened HAUS5-correlated genes based on ccRCC transcriptome data. GO enrichment analysis was adopted to interpret the biological processes enriched in these genes, and GSEA further uncovered pathway variations across distinct HAUS5 expression phenotypes. Pearson correlation analysis was used to examine the relationship between HAUS5 expression and molecules involved in MAPK/ERK signaling or epithelial-mesenchymal transition (EMT).

### Cell culture and transfection

Renal cancer cell lines and HK-2 normal kidney epithelial cells were incubated under conventional cultivation environments. HAUS5 knockdown and overexpression were performed in the indicated ccRCC cell lines according to the experimental design. After transfection or stable cell-line construction, cells with altered HAUS5 expression were collected for molecular detection and phenotype-related assays.

### Reagent treatment

EGF was used at 50 ng/mL in rescue experiments, in which ACHN cells were analyzed in four parallel groups: shNC, shNC + EGF, shHAUS5, and shHAUS5 + EGF. Belinostat was applied at the corresponding IC50 concentration determined for each cell line, and DMSO was used as the vehicle control. Cells receiving the indicated interventions were further subjected to molecular profiling and functional validation assays.

### IHC, qRT-PCR, and western blotting

Immunohistochemical (IHC) staining was performed on clinical specimens and xenograft tumor sections using antibodies against HAUS5, Ki67, E-cadherin, N-cadherin, Snail, and Vimentin. For qRT-PCR, total RNA was extracted from tissues or cells, reverse-transcribed into cDNA, and quantified using GAPDH as the internal reference. Western blot bands were quantified using ImageJ software. Protein expression levels were normalized to β-actin, whereas phosphorylated ERK1/2 levels were normalized to total ERK1/2. Quantification was performed from at least three independent biological experiments.

### Cell functional assays

Cell proliferative phenotypes were verified using CCK-8 and EdU staining, with colony formation assays adopted to assess sustained cellular growth capacity. Cell migration was examined using wound-healing assays, and Transwell assays with Matrigel-coated upper chambers were used to assess invasion. Before these assays, viable cells from each experimental group were counted using an automated cell counter and adjusted to the same concentration to ensure identical initial cell numbers. Both assays were performed in three independent experiments.

### MIF ELISA assay

Conditioned media were collected from ccRCC cells with different HAUS5 expression levels. MIF secretion levels were measured using a human MIF ELISA kit according to the manufacturer's instructions. The relative MIF secretion was normalized to the corresponding control group. Three independent experiments were performed.

### Macrophage-related assays

THP-1 monocytes were differentiated into macrophage-like cells following PMA induction. To evaluate the involvement of MIF signaling, OE-HAUS5 ccRCC cells were treated with the MIF inhibitor ISO-1 (5 μM) for 24 h. Conditioned media were collected from OE-NC cells, OE-HAUS5 cells, and ISO-1-treated OE-HAUS5 cells, centrifuged to remove cell debris, and subsequently applied to THP-1-derived macrophages. Macrophage recruitment was assessed using Transwell assays. Phagocytosis and macrophage polarization were analyzed by flow cytometry using fluorescent substrates and antibodies against CD86 and CD206.

### Xenograft model

All procedures involving BALB/c nude mice were reviewed and approved by the Institutional Animal Ethics Committee of Yantai Yuhuangding Hospital (Approval No K2026–246) and were conducted in accordance with institutional guidelines for the care and use of laboratory animals. Male BALB/c nude mice were inoculated with stably modified ACHN cell lines (shHAUS5 and shNC) to establish subcutaneous tumor models. Continuous measurements of body weight and tumor parameters were performed throughout the study. Tumor volumetric analysis was conducted using the equation: length × width² / 2. All tumor specimens were harvested, imaged and weighed post-experimentally for further IHC staining and analysis.

### Drug sensitivity and molecular docking

Drug sensitivity correlation analysis was performed to identify HAUS5-related candidate compounds. CCK-8 assay results were used to fit dose-response curves and determine the IC50 of Belinostat. Structural data of HAUS5 (PDB ID: 7SQK) was sourced from PDB, while small molecule models were obtained from PubChem. Docking was performed using AutoDock Vina 1.2.3 after receptor and ligand preparation with PyMOL 2.5.5 and ADFRsuite 1.0. The conformation with the lowest predicted docking energy was selected for graphical visualization.

### Statistical analysis

Statistical analyses were performed using GraphPad Prism and R software. Quantitative data are presented as the mean ± standard deviation (SD). Normality of continuous variables was assessed using the Shapiro–Wilk test before parametric analyses. For comparisons between two independent groups, a two-tailed unpaired Student’s *t*-test was used for normally distributed data, whereas the Mann–Whitney U test was used for non-normally distributed data. For paired tumor and adjacent non-tumor samples, a paired Student’s *t*-test was used when the paired differences were normally distributed; otherwise, the Wilcoxon signed-rank test was applied. For comparisons among three or more groups, one-way ANOVA followed by Tukey’s multiple-comparisons test was used for normally distributed data, whereas the Kruskal–Wallis test followed by Dunn’s multiple-comparisons test was used for non-normally distributed data. For experiments involving both group and time factors, including CCK-8 assays and longitudinal tumor-growth measurements, two-way ANOVA was performed, with multiple-comparisons correction where appropriate. Kaplan–Meier survival curves were compared using the log-rank test, and Cox proportional-hazards regression was used for survival-risk analysis. Pearson correlation analysis was used for continuous variables showing approximately normal distributions and linear relationships; otherwise, Spearman rank correlation analysis was applied. All statistical tests were two-sided, and P < 0.05 was considered statistically significant.

## Funding statement

The author(s) declare financial support was received for the research and/or publication of this article. This work was supported by the 10.13039/501100001809National Natural Science Foundation of China (Nos. 82370690, 82073131, and 82270722), the 10.13039/501100007129Natural Science Foundation of Shandong Province (No. ZR2025MS1283), the Taishan Scholars Project of Shandong Province (No. tsqn202408401), and Postdoctoral Innovation Program of Shandong Province (No. SDCX-ZG-202501046).

## CRediT authorship contribution statement

**Youwei Chi:** Visualization, Validation, Methodology, Investigation, Formal analysis, Data curation, Conceptualization. **Yanfei Li:** Formal analysis, Data curation. **Bin Yang:** Supervision. **Guixin Ding:** Methodology. **Chao Tang:** Validation. **Fengze Sun:** Resources. **Qian Li:** Supervision. **Renxiang Zhang:** Methodology. **Jian Ma:** Data curation. **Hongchang Zhang:** Conceptualization. **Jitao Wu:** Conceptualization. **Yongwei Li:** Project administration, Methodology, Investigation, Funding acquisition, Formal analysis.

## Declaration of competing interest

The authors declare that they have no known competing financial interests or personal relationships that could have appeared to influence the work reported in this paper.

## Data Availability

Data are available upon reasonable request. The datasets used and/or analyzed during this study are available from the corresponding author upon reasonable request.
