# Supplementary material for: HAUS5 defines a mitogen-activated protein kinase/extracellular signal-regulated kinase-dependent epithelial–mesenchymal plasticity program and therapeutic vulnerability to Belinostat in clear cell renal cell carcinoma
Source: Transl Oncol. 2026 Nov 6;75:103107. doi: 10.1016/j.tranon.2026.103107 (PMC13769121; doi:10.1016/j.tranon.2026.103107)

Fig.2C

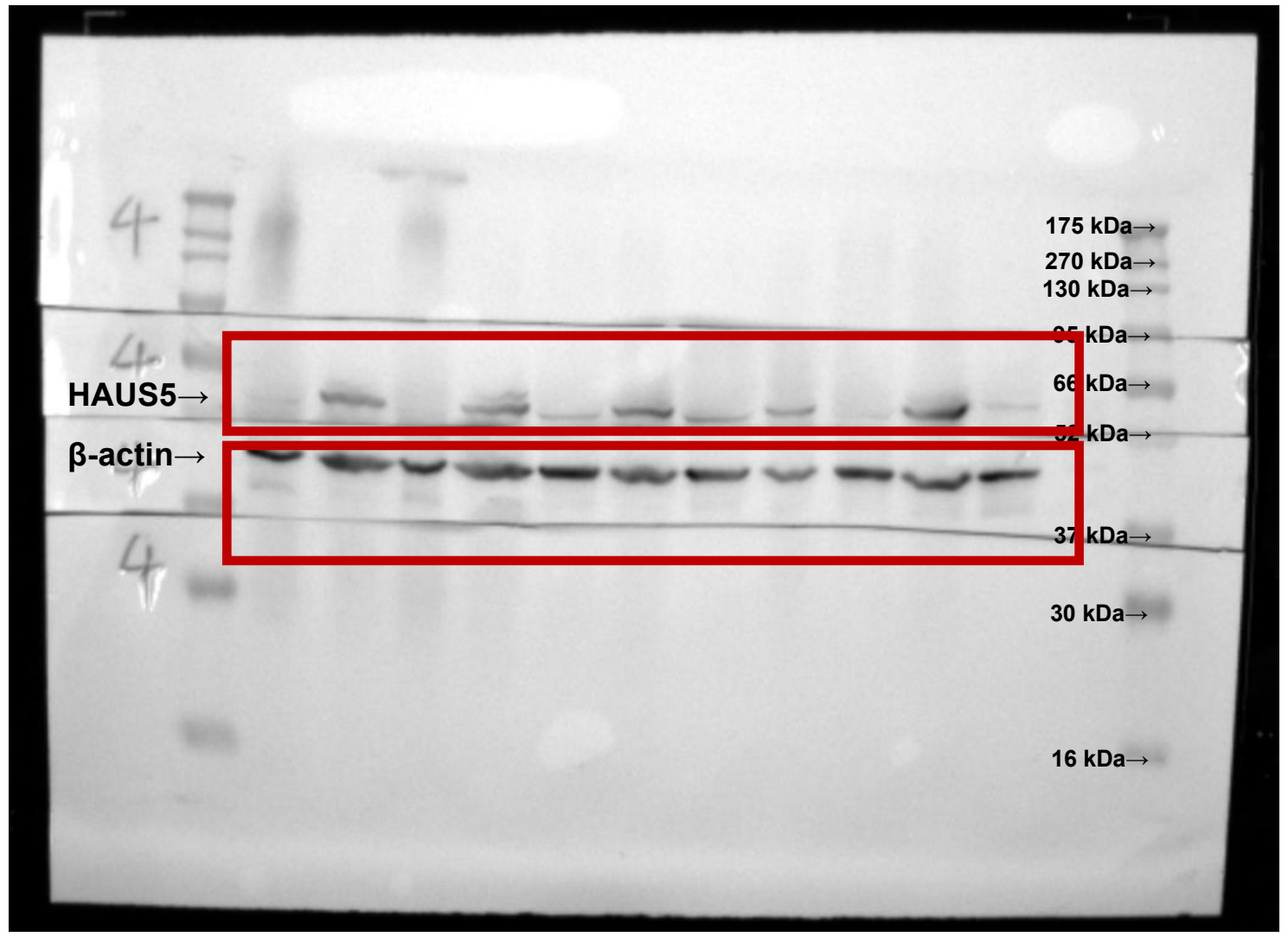

Fig.2D - Repeat 1

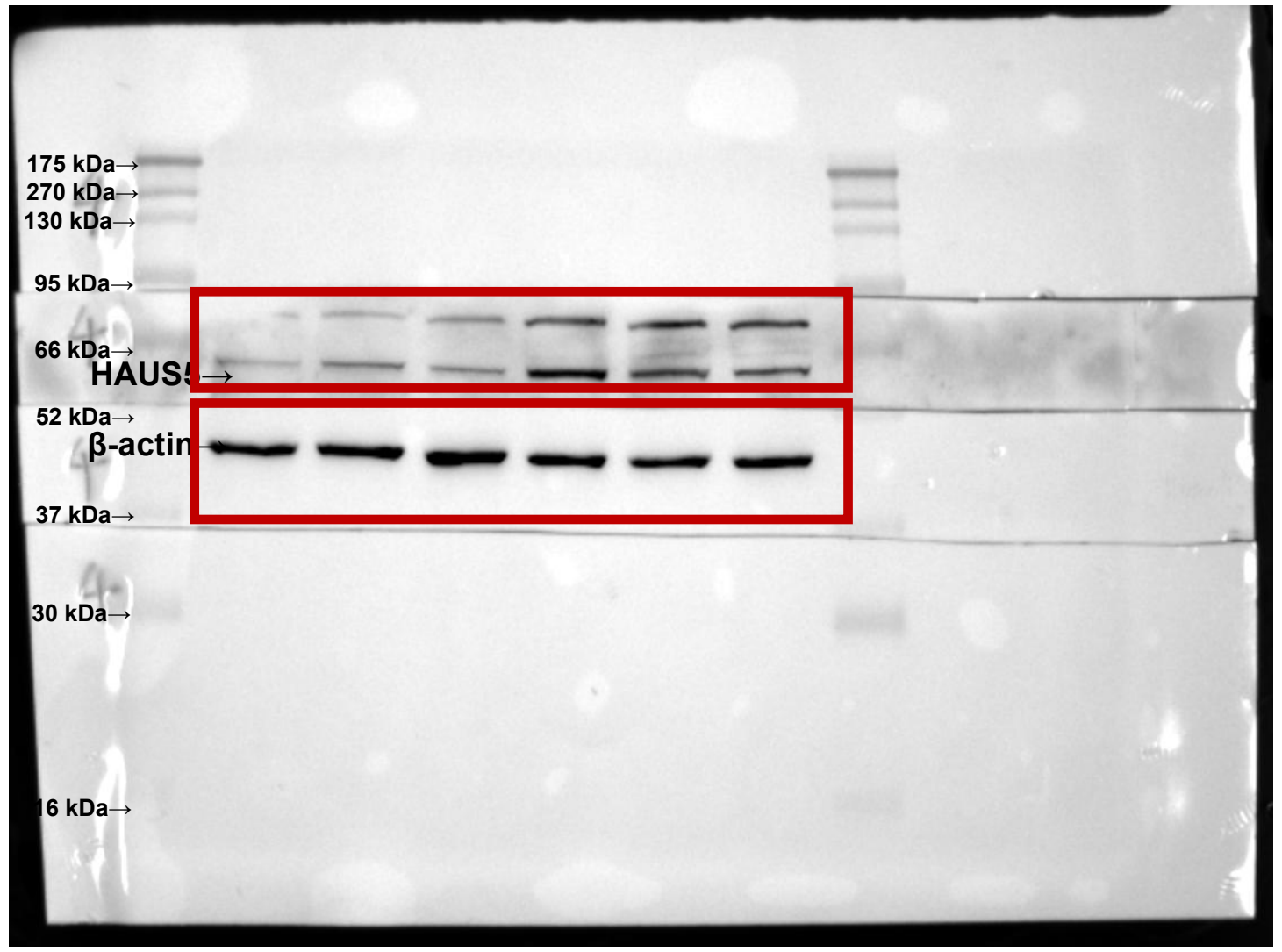

Fig.2D - Repeat 2

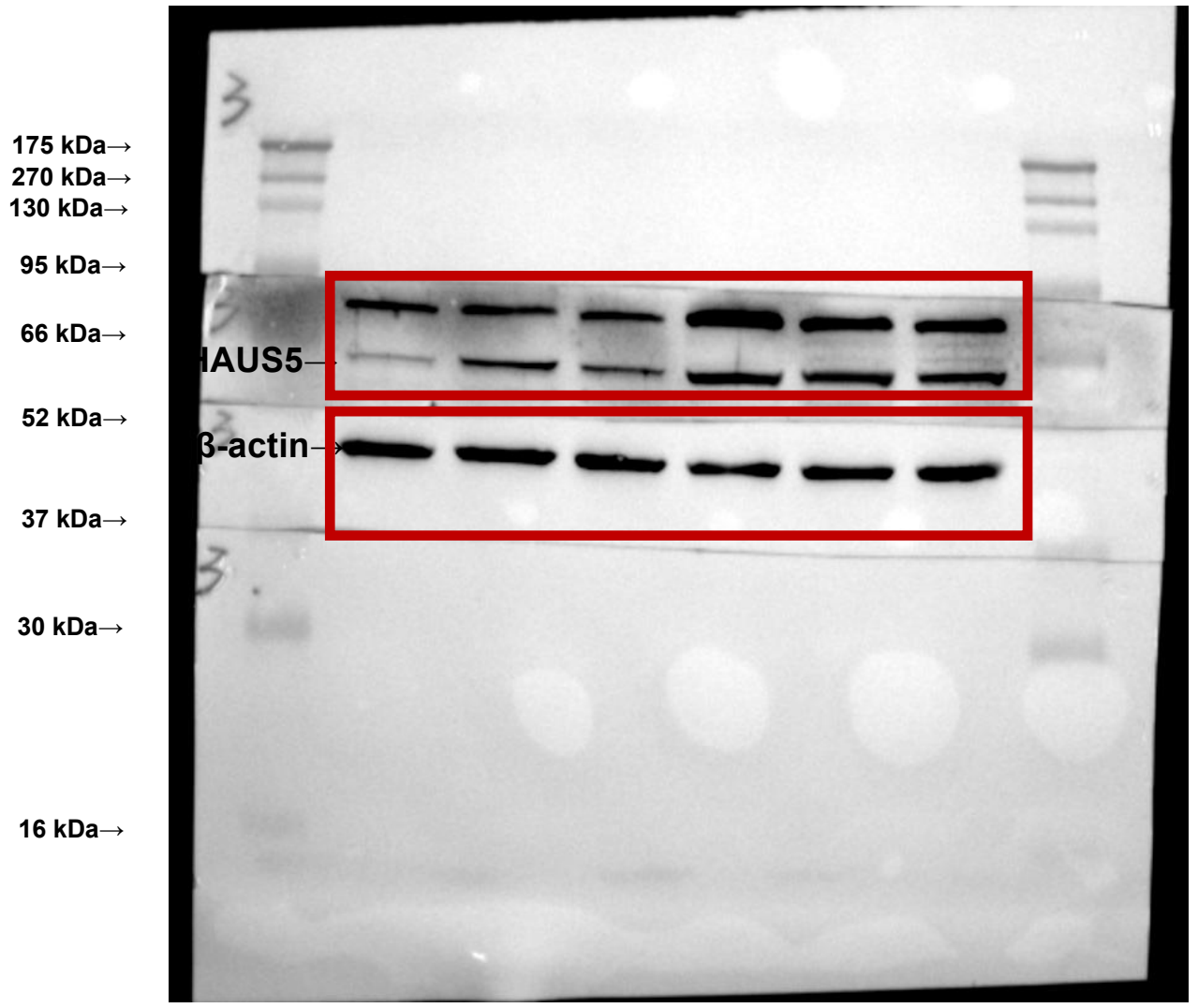

Fig.2D - Repeat 3

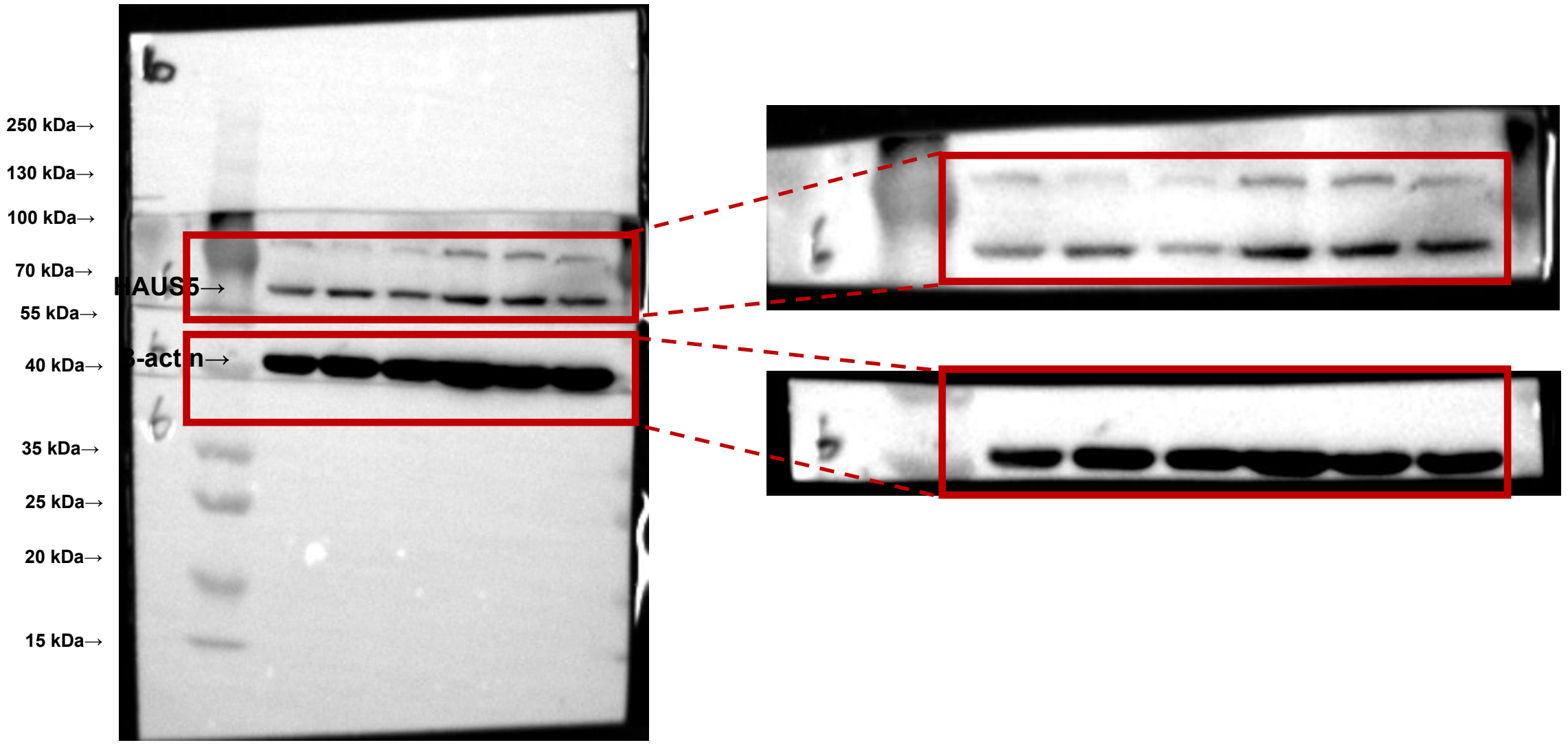

Fig.2G

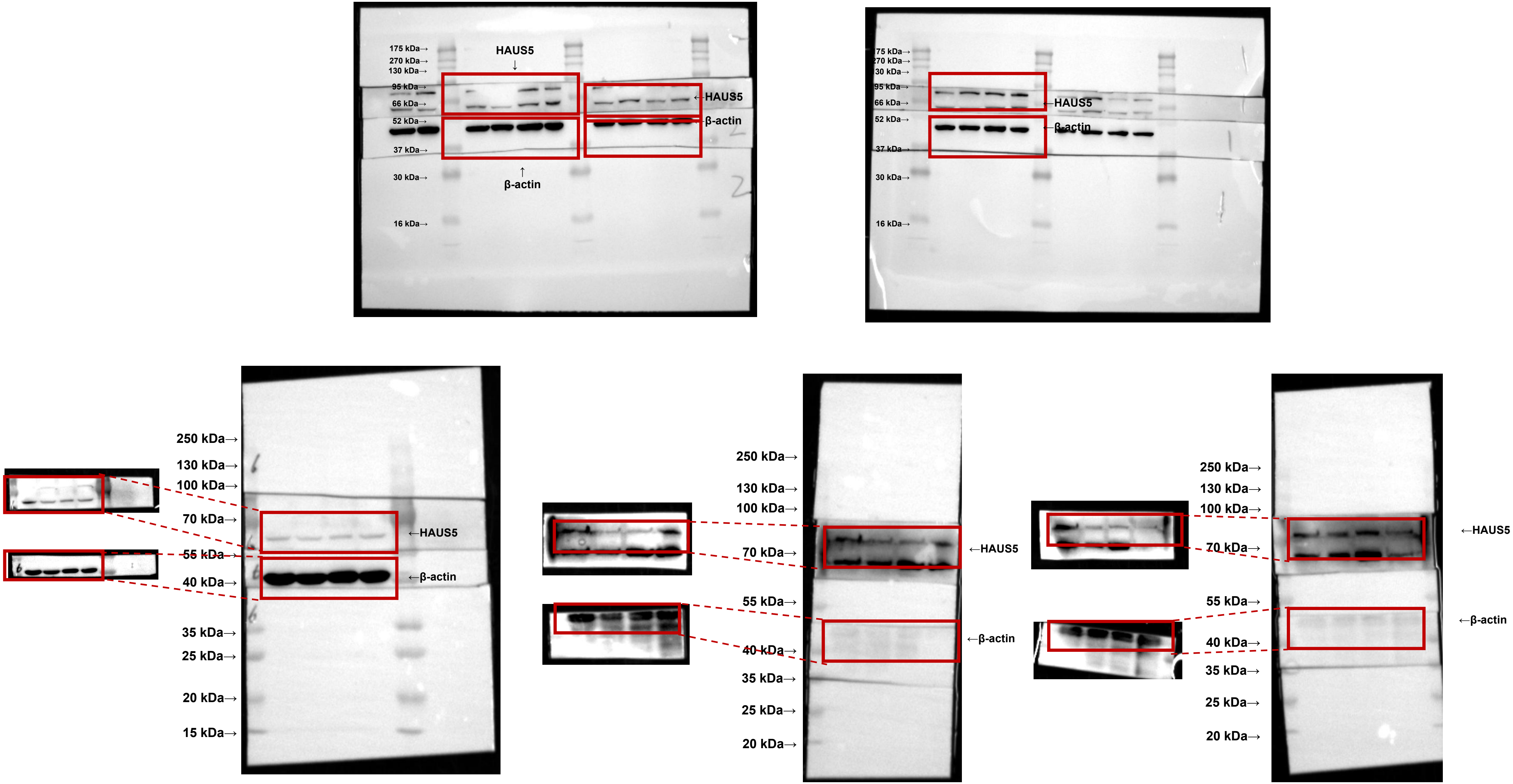

175 kDa→
270 kDa→
130 kDa→
95 kDa→
66 kDa→
52 kDa→
37 kDa→
30 kDa→
16 kDa→
HAUS5
β-actin
←HAUS5
←β-actin
250 kDa→
130 kDa→
100 kDa→
70 kDa→
55 kDa→
40 kDa→
35 kDa→
25 kDa→
20 kDa→
15 kDa→

Fig.4G

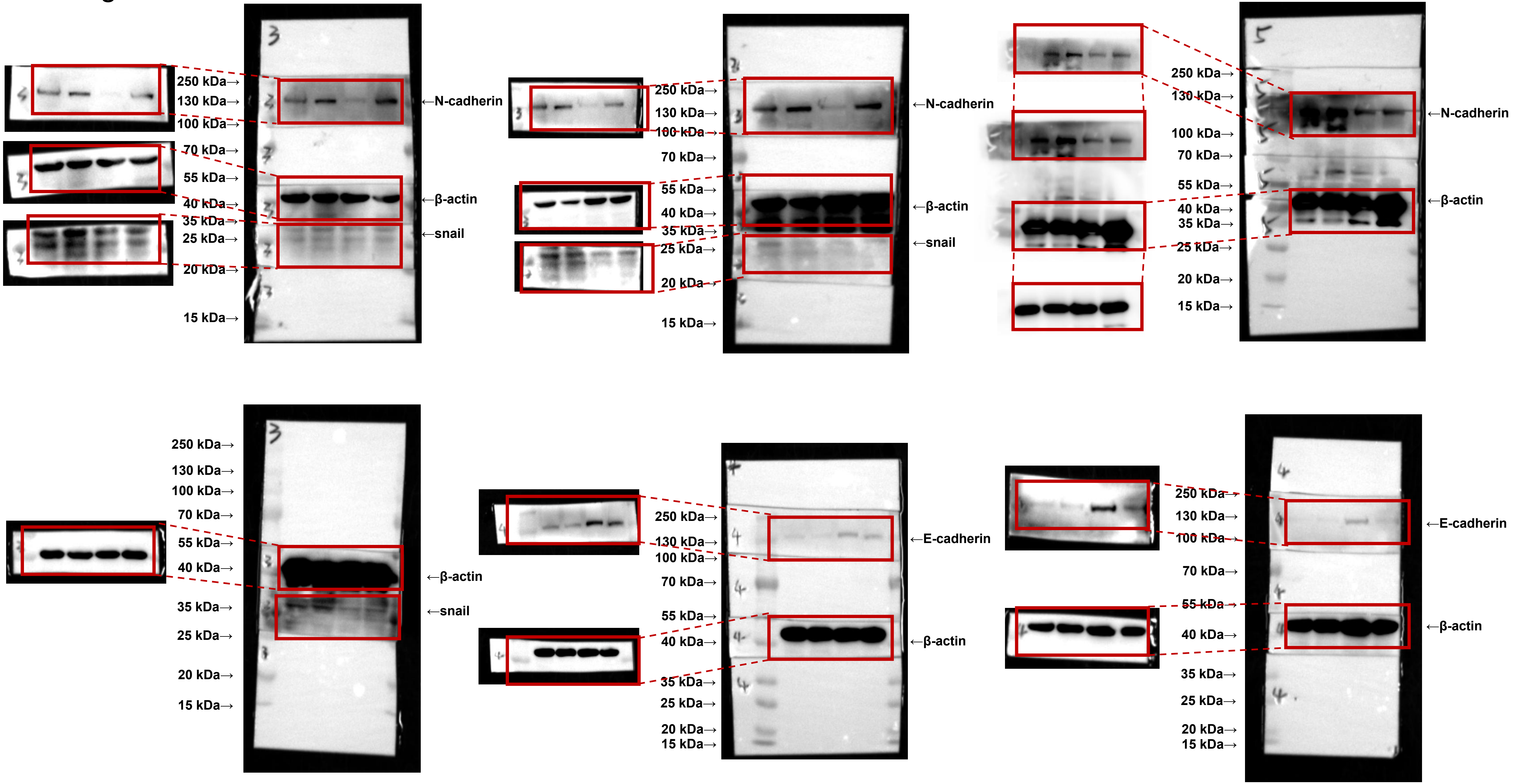

250 kDa→
130 kDa→
100 kDa→
70 kDa→
55 kDa→
40 kDa→
35 kDa→
25 kDa→
20 kDa→
15 kDa→
←N-cadherin
←β-actin
←snail
←E-cadherin

Fig.4G

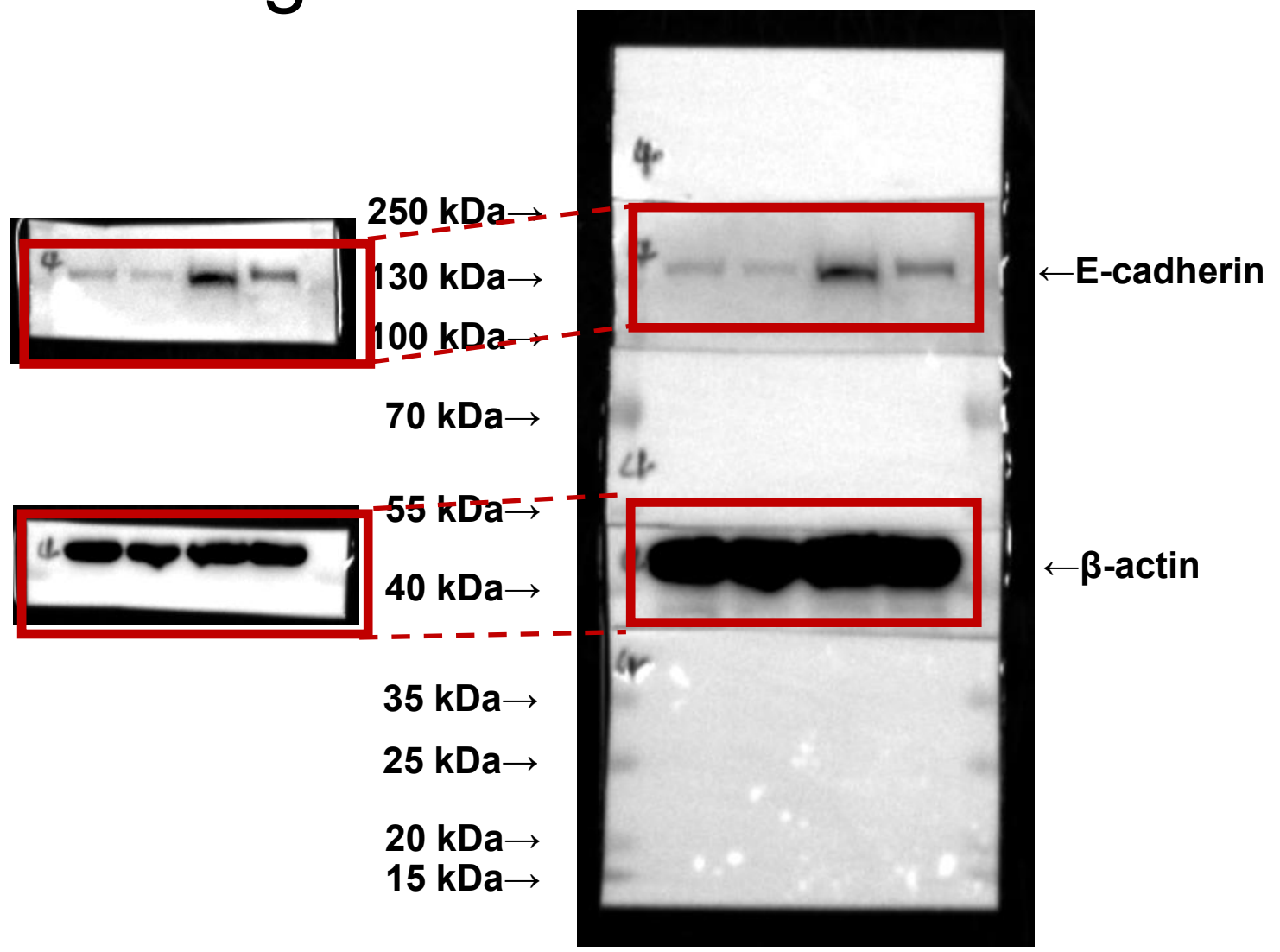

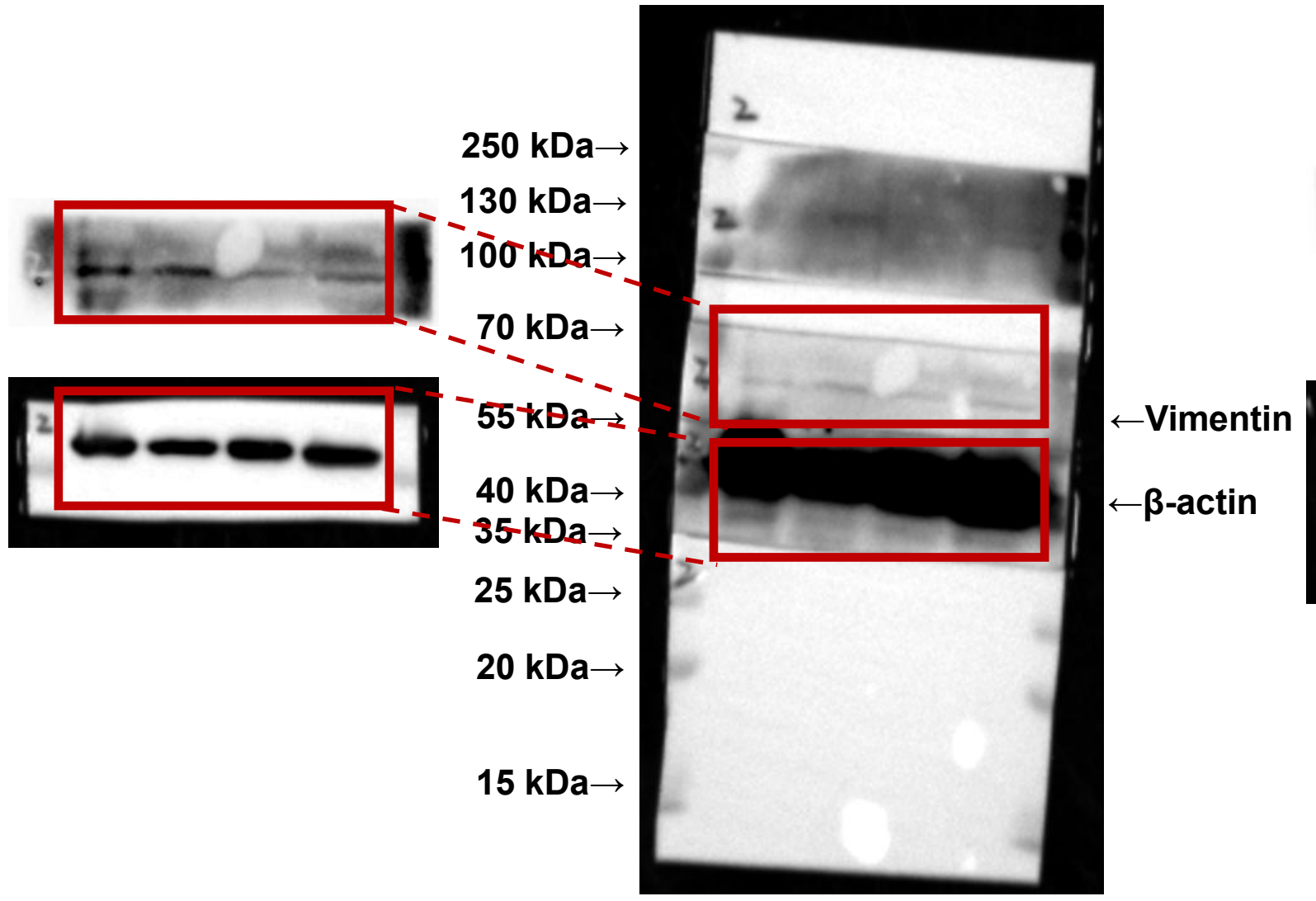

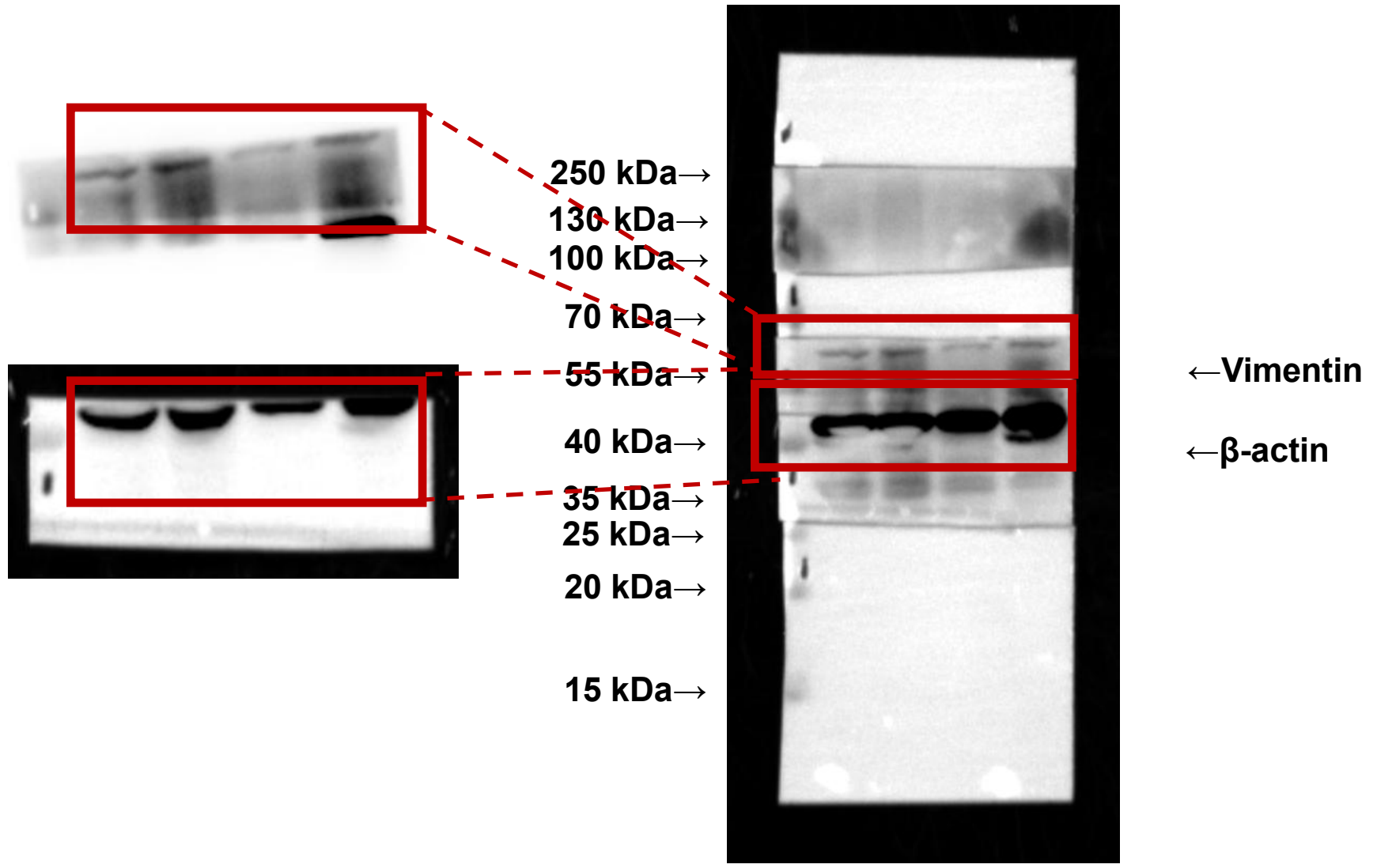

Fig.4H - Repeat1

Fig.4H - Repeat2

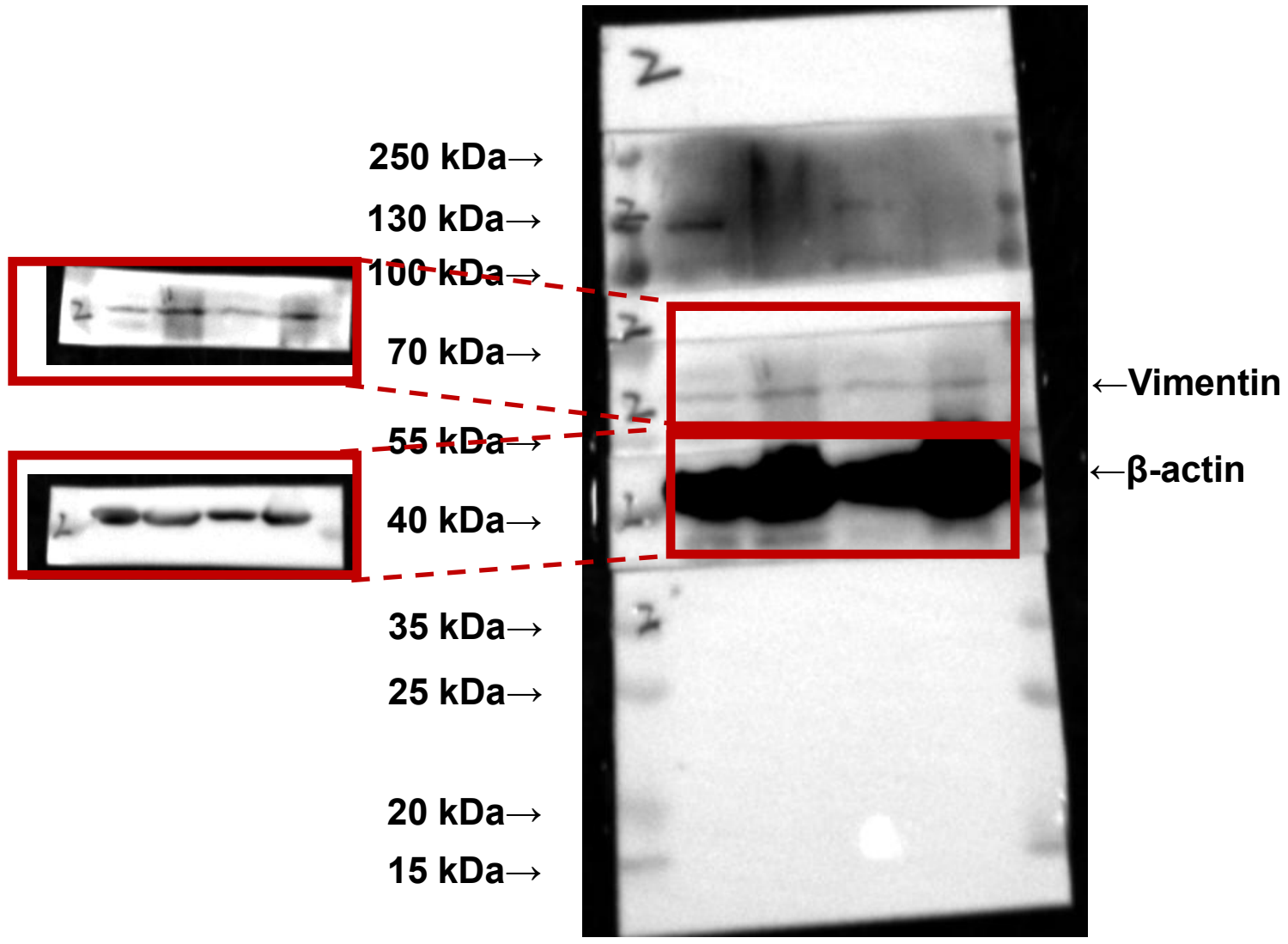

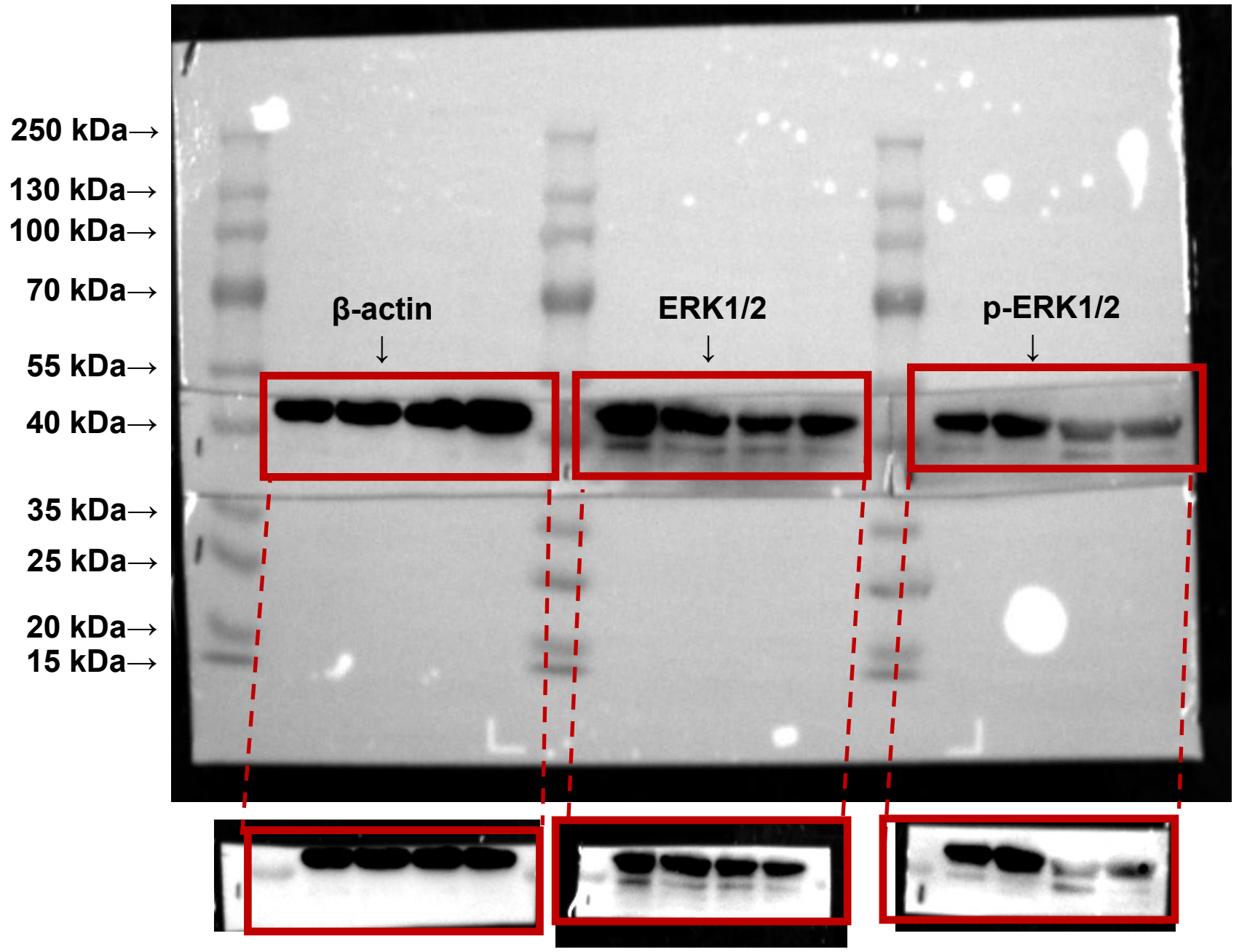

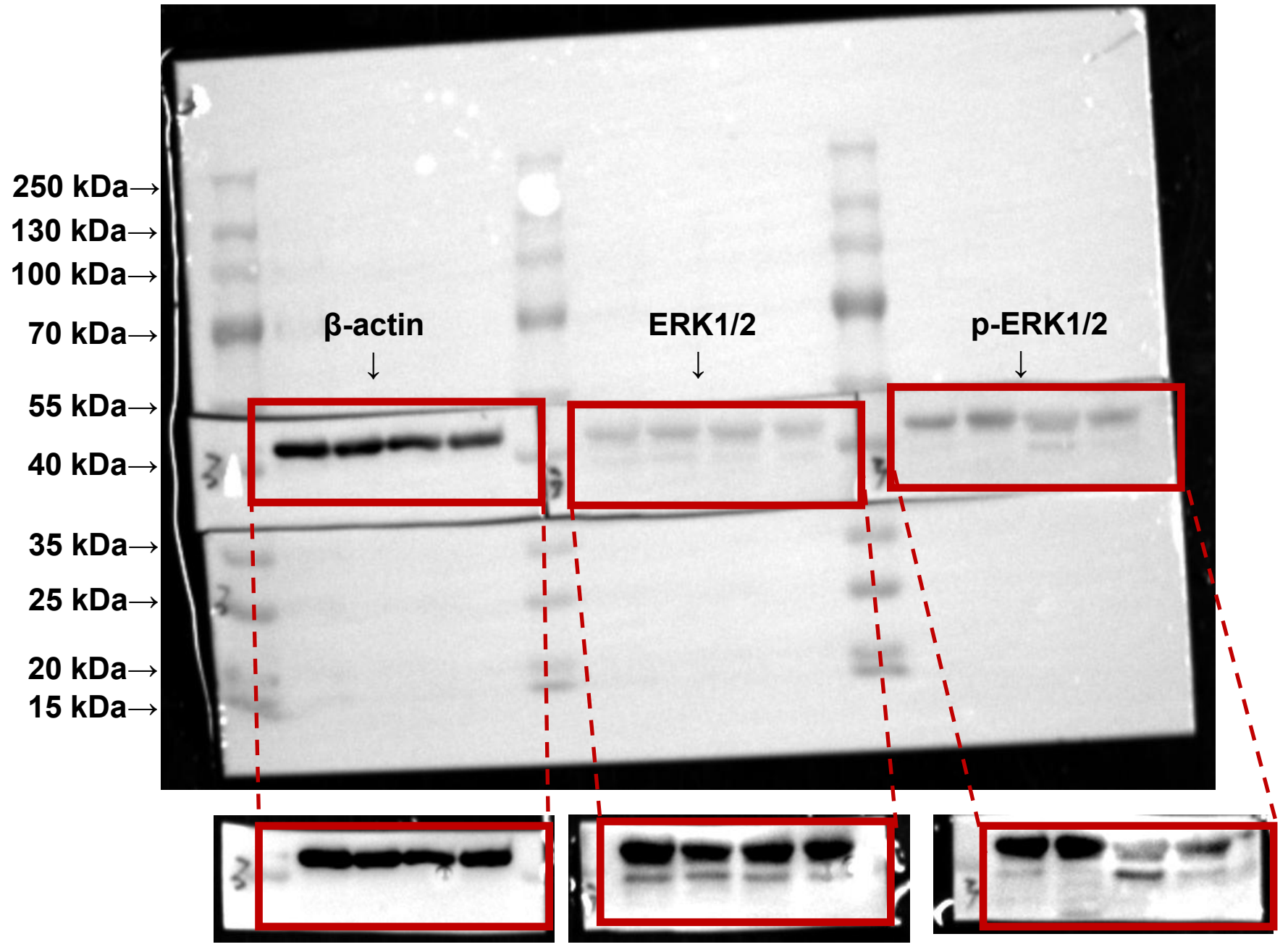

Fig.4H - Repeat3

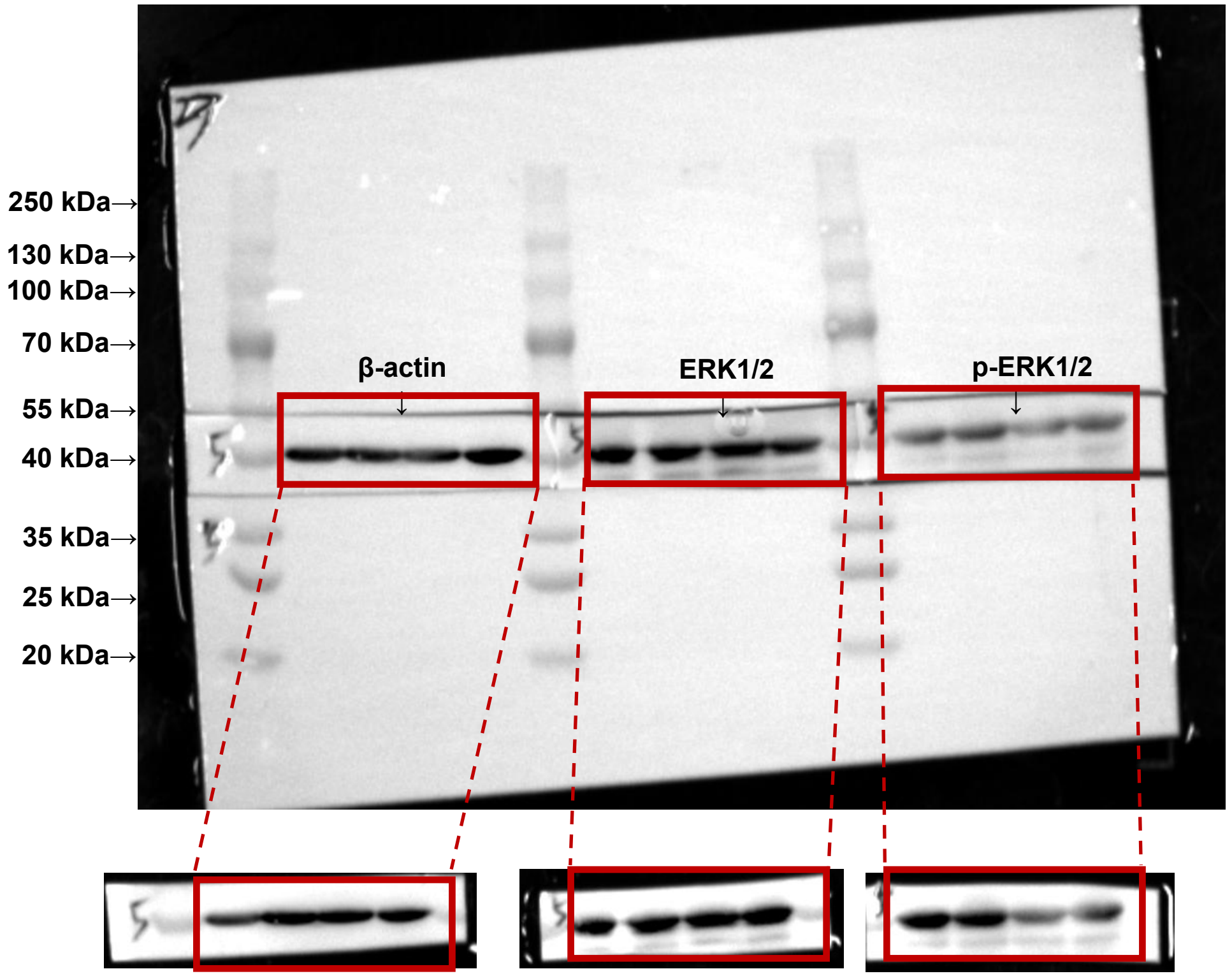

Supplement: Supplementary file 1 [file mmc1.pdf]
