# Supplementary material for: HAUS5 defines a mitogen-activated protein kinase/extracellular signal-regulated kinase-dependent epithelial–mesenchymal plasticity program and therapeutic vulnerability to Belinostat in clear cell renal cell carcinoma
Source: Transl Oncol. 2026 Nov 6;75:103107. doi: 10.1016/j.tranon.2026.103107 (PMC13769121; doi:10.1016/j.tranon.2026.103107)
Supplement: Supplementary file 2 [file mmc2.docx]

**Supplementary Fig. 1**

**Effects of HAUS5 modulation on cell proliferation and MIF secretion in ccRCC cells.**

(A) CCK-8 assay showing the proliferation of ACHN cells under four conditions: shNC, shNC + EGF, shHAUS5, and shHAUS5 + EGF at the indicated time points. (B) Relative MIF concentration in conditioned medium from ACHN cells with HAUS5 knockdown compared with the shNC group. (C) Relative MIF concentration in conditioned medium from 769P cells with HAUS5 overexpression compared with the OE-NC group. Data are presented as mean ± SD from three independent experiments. ns, not significant; *P < 0.05, ***P < 0.001, ****P < 0.0001.

**Supplementary Table 1**

Basic clinicopathological characteristics of the 12 patients with clear cell renal cell carcinoma included in the in-house cohort.

| **Items** | **ccRCC patients (N = 12)** |
| --- | --- |
| Age (y) | 59.6 ± 10.8 |
| Gender |  |
| Male (n/%) | 7 (58.3) |
| Female (n/%) | 5 (41.7) |
| Tumor location |  |
| Right (n/%) | 6 (50.0) |
| Left (n/%) | 6 (50.0) |
| Pathological grade |  |
| 1 (n/%) | 4 (33.3) |
| 2 (n/%) | 6 (50.0) |
| 3 (n/%) | 2 (16.7) |
| Tumor size (cm) | 4.8 (3.9–6.5) |
| T stage |  |
| T1 (n/%) | 8 (66.7) |
| T2 (n/%) | 3 (25) |
| T3 (n/%) | 1 (8.3) |
| N stage |  |
| N0 (n/%) | 11 (91.7) |
| N1 (n/%) | 1 (8.3) |
| TNM stage |  |
| I (n/%) | 8 (66.7) |
| II (n/%) | 3 (25) |
| III (n/%) | 1 (8.3) |
